# LOXL1, THY1, and TYMS define an annotation-derived hemoglobin-associated immunotranscriptomic signature in osteoarthritis cartilage

**DOI:** 10.3389/fimmu.2026.1823558

**Published:** 2026-07-09

**Authors:** Xin Zhang, Zhaochen Ye, Zheng Qin, Yizhe Li, Zhilong Jing, Dawei Yang

**Affiliations:** Department of Orthopedics, The Fourth Affiliated Hospital of Harbin Medical University, Harbin, China

**Keywords:** cartilage biomarkers, hemoglobin-associated annotation, immunotranscriptomic signature, machine learning, osteoarthritis, WGCNA

## Abstract

**Background:**

Osteoarthritis (OA) is increasingly recognized as a whole-joint disease involving cartilage degeneration, synovial inflammation, subchondral bone remodeling, and immune-associated inflammatory signaling. Non-erythroid hemoglobin (Hb)-associated transcriptional programs have recently been implicated in chondrocyte hypoxic adaptation, but their relationship with immune-associated transcriptomic remodeling in OA cartilage remains unclear. This study aimed to identify annotation-derived Hb-associated transcriptomic biomarkers in OA cartilage and to characterize their associations with inferred immune-related transcriptomic features.

**Methods:**

Human cartilage transcriptomic datasets were integrated from Gene Expression Omnibus (GEO) to construct a harmonized training cohort, with independent datasets used for external validation. After batch-effect correction, OA differentially expressed genes were intersected with a GeneCards-derived Hb-associated annotation gene set. Weighted gene co-expression network analysis, together with the least absolute shrinkage and selection operator and other complementary machine-learning approaches, was applied to identify hub Hb-associated OA genes. A multivariable logistic-regression nomogram was constructed and evaluated by receiver operating characteristic analysis across cohorts. Immune pathway activity and immune-cell-associated transcriptomic signatures were inferred using GSVA, CIBERSORT, ssGSEA, and correlation analyses. Experimental validation was performed using qRT-PCR in an interleukin-1β-stimulated inflammatory chondrocyte model.

**Results:**

Three Hb-associated hub genes—LOXL1, THY1, and TYMS—were identified through integrative transcriptomic analysis and consistently upregulated in OA cartilage across training and independent validation cohorts. Immune profiling suggested an immune-activated transcriptomic state in OA cartilage, characterized by increased macrophage-associated signals, reduced resting NK and CD4 memory T cells, elevated activated immune cell signatures, and enhanced antigen presentation-related and immune regulation-related functions. Expression of these Hb-associated hub genes showed strong positive correlations with macrophage- and activated T-cell-related signatures and antigen presentation-associated immune functions. A nomogram based on the three genes demonstrated high discriminative performance across cohorts, with an AUC of 0.974 in the training cohort. qRT-PCR in an IL-1β-stimulated C28/I2 chondrocyte model supported the inflammatory inducibility of LOXL1, THY1, and TYMS.

**Conclusion:**

This study identifies LOXL1, THY1, and TYMS as reproducible annotation-derived Hb-associated tissue-level transcriptomic biomarkers in OA cartilage. Their expression is associated with macrophage- and activated T-cell-related transcriptomic signatures, providing an immunotranscriptomic link between annotation-derived Hb-associated gene programs and immune remodeling in OA.

## 1 Introduction

Osteoarthritis (OA) is one of the most prevalent degenerative joint diseases and a leading cause of chronic pain, disability, and reduced quality of life in the aging population (1). Although traditionally viewed as a mechanically driven disorder, OA is now increasingly recognized as a chronic inflammatory joint disease in which immune activation within the joint microenvironment contributes to cartilage degeneration and disease progression (2, 3). Pathologically, OA is characterized by progressive articular cartilage breakdown accompanied by low-grade synovial inflammation, subchondral bone remodeling, and alterations in local immune cell composition and signaling (4, 5). While mechanical loading, metabolic dysregulation, and cellular senescence are important contributors to OA pathogenesis, accumulating evidence suggests that immune and inflammatory processes contribute to OA initiation and progression (6). However, clinically applicable molecular biomarkers that integrate immune dysregulation with chondrocyte biology in OA cartilage remain limited (7).

Articular cartilage is an avascular tissue in which resident chondrocytes must adapt to persistent hypoxic conditions, shaping cellular metabolism, stress responses, and inflammatory signaling (8, 9). Recent transcriptomic studies, including single-cell RNA sequencing analyses, have revealed that non-erythroid hemoglobin (Hb) genes have been detected in specific chondrocyte subpopulations and exhibit distinct expression patterns between healthy and osteoarthritic cartilage (10, 11). These Hb-associated transcriptional differences have been linked to altered signaling pathways and intercellular communication within the OA microenvironment (12). Recent evidence further suggests that chondrocytes may use non-erythroid Hb-associated programs, including Hb-body formation, as part of their adaptation to hypoxic stress (13). Notably, hypoxia and oxidative stress are increasingly recognized as key modulators of immune activation and inflammatory responses (14, 15). Together, these findings provide a rationale for exploring whether Hb-associated annotation-defined genes are linked to immune-related transcriptomic remodeling in OA cartilage.

**Figure 1:**
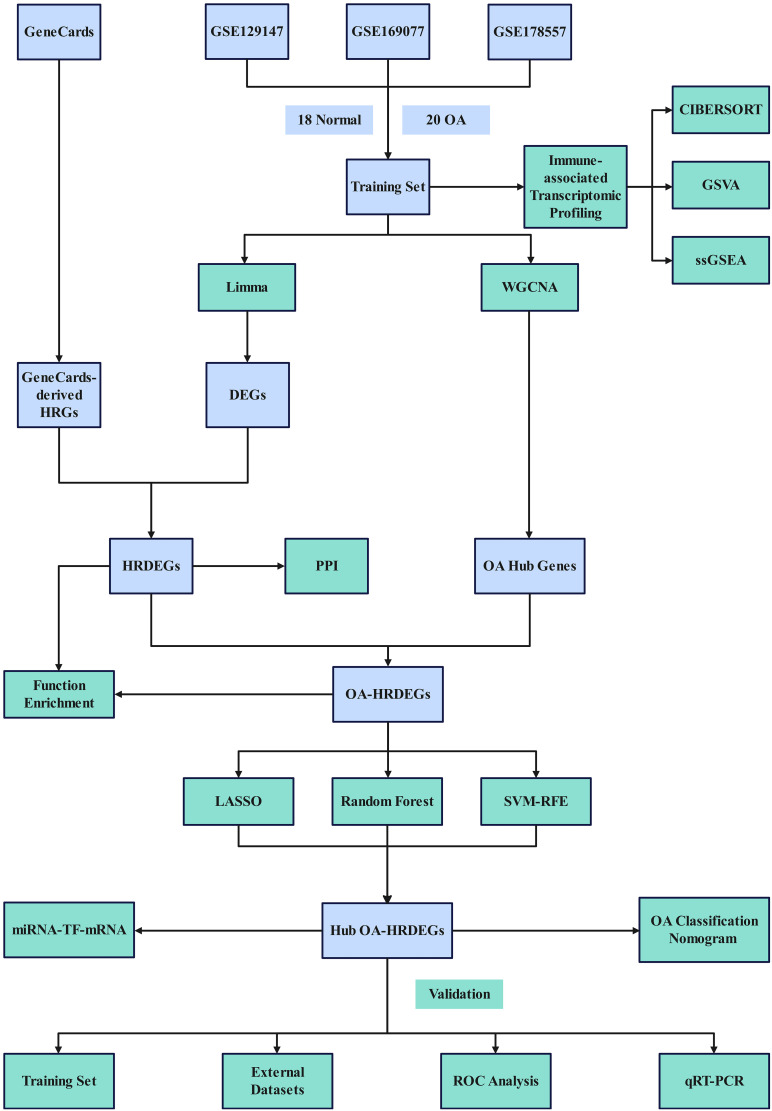
Overview of the integrative analytical workflow used in this study.

Based on these observations, we hypothesized that Hb-associated annotation-defined transcriptional programs may be associated with inflammatory signaling and immune-related transcriptomic features in OA cartilage. To test this hypothesis, we integrated differential gene expression analysis, weighted gene co-expression network analysis, and curated HRGs across multiple GEO cartilage transcriptomic datasets to define OA-associated Hb-related differentially expressed genes (OA-HRDEGs). Complementary machine-learning algorithms were applied to identify robust hub OA-HRDEGs with diagnostic potential, and a nomogram-based classification model was constructed and validated in independent cohorts. In parallel, immune-related pathway activity and immune-cell-associated transcriptomic signatures were characterized using gene set variation analysis (GSVA), CIBERSORT, and single-sample gene set enrichment analysis (ssGSEA) to delineate immune landscapes associated with these hub genes. Finally, candidate biomarkers were experimentally validated by qRT-PCR in an IL-1β-stimulated inflammatory chondrocyte model. Together, this integrative, multi-level approach provides new insight into the interplay between Hb-associated gene programs and immune-mediated processes in OA cartilage, with implications for tissue-level biomarker classification and hypothesis generation for future mechanistic studies. The overall workflow of the study is illustrated in **Figure 1**.

## 2 Materials and methods

### 2.1 Data download and processing

OA-related cartilage transcriptomic datasets were retrieved from the Gene Expression Omnibus (GEO) database. Six independent GEO datasets derived from human articular cartilage were included in this study. Among them, three datasets (GSE129147, GSE169077, and GSE178557) were designated as the training cohort for model development, while the remaining datasets (GSE113825 and GSE114007 generated on two different platforms) were used as independent external validation cohorts.

To construct the training cohort, genes commonly expressed across the three training datasets were identified and integrated into a unified expression matrix. To minimize technical variability arising from differences in experimental platforms and batch effects, expression data from the training cohort were normalized and corrected using the ComBat algorithm implemented in the sva R package (16). The effectiveness of batch-effect correction was assessed by visual comparison of gene expression distributions before and after correction. Detailed information regarding dataset characteristics, platform annotation, and sample composition was provided in **Table 1**.

**Table 1:** The datasets from the GEO database.

GSE	GPL	Species	Tissue source	Sample number		Attribute
				OA	Control	
GSE129147	GPL15207	Homo sapiens	Cartilage	10	9	Training
GSE169077	GPL96	Homo sapiens	Cartilage	6	5	Training
GSE178557	GPL13497	Homo sapiens	Cartilage	4	4	Training
GSE113825	GPL22516	Homo sapiens	Cartilage	5	5	Validation
GSE114007	GPL11154	Homo sapiens	Cartilage	10	8	Validation
GSE114007	GPL18573	Homo sapiens	Cartilage	10	10	Validation

### 2.2 Identification of DEGs

Following batch-effect correction and data normalization, differential expression analysis was performed to identify genes differentially expressed between OA and control cartilage samples in the training cohort. The limma R package was applied to fit linear models for each gene, with empirical Bayes moderation used to improve variance estimation (17). Genes meeting the criteria of *p* ≤ 0.05 and |log_2_ fold change| ≥ 0.585 were defined as differentially expressed genes (DEGs) and retained for subsequent analyses.

The distribution of DEGs was visualized using volcano plots, and hierarchical clustering heatmaps of representative DEGs were generated to illustrate expression patterns between OA and control samples. All visualizations were performed using standard R packages, including ggplot2 and pheatmap, with row-wise Z-score normalization applied where appropriate.

### 2.3 Compilation and operational definition of HRGs

A comprehensive set of Hb-related genes (HRGs) was compiled for systems-level analysis by retrieving genes from the GeneCards database using the keyword “Hemoglobin.” GeneCards integrates gene–disease and gene–function associations from multiple curated sources (18). In the present study, HRGs were operationally defined as GeneCards-derived Hb-associated genes, representing a broad Hb-associated annotation space rather than a canonical hemoglobin-subunit gene set. After removal of duplicate entries, a total of 16,128 genes were retained as the reference HRG set for subsequent integrative analyses. No GeneCards relevance-score cutoff was applied in the primary analysis, in order to maximize sensitivity for detecting genes directly or indirectly annotated to Hb-related oxygen handling, hypoxia adaptation, oxidative stress, erythroid-associated biology, or immune-related processes. Therefore, throughout this study, the term “Hb-associated” refers to GeneCards annotation-derived association and does not imply that the identified hub genes encode hemoglobin subunits. The complete list of HRGs was provided in **Supplementary Table 1**.

### 2.4 Identification of HRDEGs

Hb-related differentially expressed genes (HRDEGs) were identified by intersecting OA-associated DEGs with the GeneCards-derived HRG set defined above.

### 2.5 Construction of PPI networks

To evaluate potential interactions among HRDEGs at the protein level, protein-protein interaction (PPI) networks were constructed using the STRING database (19). Only interactions with a confidence score greater than 0.4 were retained to ensure moderate interaction reliability for subsequent network analyses (20).

### 2.6 WGCNA analysis for identification of hub genes

Weighted gene co-expression network analysis (WGCNA) was performed in the training cohort using the WGCNA R package to identify gene co-expression modules associated with OA (21, 22). To reduce noise and computational burden, the top 50% most variable genes, corresponding to 11,836 genes, were selected for network construction (23). An appropriate soft-thresholding power was determined to ensure approximate scale-free topology, and a signed weighted adjacency matrix was constructed based on Pearson correlation coefficients. The adjacency matrix was subsequently transformed into a topological overlap matrix, and gene modules were identified using hierarchical clustering combined with the dynamic tree cut algorithm.

Module-trait relationships were evaluated by correlating module eigengenes with the OA phenotype, and modules showing the strongest positive association with OA were prioritized for further analysis. Within these key modules, hub genes were identified based on module membership (MM) and gene significance (GS), with genes meeting the criteria of |MM| ≥ 0.70 and |GS| ≥ 0.30 defined as hub genes closely associated with the disease phenotype (24).

### 2.7 Identification of OA-HRDEGs

OA-associated hemoglobin-related differentially expressed genes (OA-HRDEGs) were defined as genes shared by osteoarthritis-related DEGs, OA-associated hub genes identified by WGCNA, and HRDEGs.

### 2.8 Functional enrichment analysis

Functional enrichment analyses were performed for both HRDEGs and OA-HRDEGs using the clusterProfiler and DOSE R packages. Gene Ontology (GO) enrichment analyses (25), including biological process (BP), cellular component (CC), and molecular function (MF) categories, as well as Kyoto Encyclopedia of Genes and Genomes (KEGG) pathway enrichment analyses (26), were conducted for both gene sets after converting gene symbols to Entrez IDs using the org.Hs.eg.db package. Enrichment terms with *p* < 0.05 and *q* < 0.05 were considered statistically significant.

Disease Ontology (DO) enrichment analysis (27) was additionally performed for HRDEGs to explore disease-associated functional patterns. For OA-HRDEGs, representative GO and KEGG enrichment results were selected for visualization, and gene-term associations were illustrated to highlight osteoarthritis-specific Hb-associated functional signatures.

### 2.9 Machine-learning-based identification of hub OA-HRDEGs

To identify the hub OA-HRDEGs, three complementary machine-learning approaches—least absolute shrinkage and selection operator (LASSO) regression (28), random forest (29), and support vector machine-recursive feature elimination (SVM-RFE) (30)—were applied for feature selection in the training cohort. LASSO logistic regression was implemented using the glmnet package to perform feature selection under L1 regularization, with the optimal penalty parameter determined by cross-validation and genes with nonzero coefficients retained. In parallel, a random forest model was constructed to capture nonlinear relationships among genes, and feature importance was evaluated based on the out-of-bag error. SVM-RFE was further applied to iteratively eliminate less informative features based on classification accuracy, with the optimal feature subset determined by cross-validation.

Feature gene sets identified by LASSO regression, random forest, and SVM-RFE were intersected to obtain the hub OA-HRDEGs, thereby enhancing the robustness and stability of feature selection. This multi-algorithm strategy is consistent with recent ensemble feature-selection frameworks that aim to reduce algorithm-specific bias and improve the stability of transcriptomic biomarker signatures (31).

### 2.10 Construction of hub OA-HRDEGs-based OA classification model

To construct an OA classification model based on hub OA-HRDEGs, the integrated training cohort comprising GSE129147, GSE169077, and GSE178557 was used. A multivariable logistic regression model was established using the rms R package, with LOXL1, THY1, and TYMS included as independent variables and disease status as the dependent variable. Model parameters were estimated using the maximum likelihood method, and a nomogram was generated to provide a visual representation of the predictive contribution of each gene and to visualize the relative contribution of each gene to sample-level OA classification (32).

Predicted probabilities derived from the fitted logistic regression model were calculated for each sample and used as model-derived OA classification scores. Model calibration was assessed using calibration curves based on 1,000 bootstrap resamples. Model discrimination was quantified using Harrell’s concordance index (C-index), with the corresponding 95% confidence interval estimated by bootstrap resampling. Decision curve analysis (DCA) was additionally performed to evaluate the exploratory net clinical benefit of the nomogram across different threshold probabilities in the training cohort. Receiver operating characteristic (ROC) curves were generated using the pROC package, and the area under the curve (AUC) with 95% confidence intervals was calculated for each individual gene and the nomogram-based model (33).

### 2.11 Validation of the OA classification model

Model performance was evaluated using internal dataset-wise validation and independent external validation. For dataset-wise validation, model performance was evaluated separately in each of the three training datasets (GSE129147, GSE169077, and GSE178557). For external validation, three independent datasets, including GSE113825 and GSE114007 generated on two platforms (GPL11154 and GPL18573), were used as external validation cohorts. For each validation dataset, nomogram-predicted probabilities were calculated using the fitted model and used as model scores; receiver operating characteristic (ROC) curves were generated using the pROC package, and area under the curve (AUC) values with corresponding 95% confidence intervals were calculated for the nomogram-based classification score and for each individual gene (LOXL1, THY1, and TYMS) to assess model stability and generalizability.

### 2.12 qRT-PCR validation of the hub OA-HRDEGs

Quantitative real-time polymerase chain reaction (qRT-PCR) validation was performed using the human chondrocyte cell line C28/I2, which was authenticated by short tandem repeat profiling (BFB Life Sciences, Shanghai, China) (34). Cells were cultured in DMEM supplemented with 10% fetal bovine serum at 37 °C in a humidified atmosphere containing 5% CO_2_. An inflammatory chondrocyte model was established by treatment with 10 ng/mL interleukin-1β (IL-1β) for 24 h, with untreated cells serving as controls (35). Three independent biological replicates were included for each group. Total RNA was extracted using TRIzol reagent, and cDNA was synthesized by reverse transcription using oligo(dT) primers. qRT-PCR was performed using a SYBR Green detection system (Roche), followed by melting curve analysis to confirm amplification specificity. Relative gene expression levels were calculated using the 2^-ΔΔCt^ method (36), and primer sequences were listed in **Table 2**.

**Table 2:** The primer sequences.

Gene	Forward primer sequence	Reverse primer sequence
LOXL1	CAGCAGACTTCCTCCCCAAC	CTGTGGTAATGCTGGTGGCA
THY1	CGGAAGACCCCAGTCCAGAT	GGGAGACCTGCAAGACTGTT
TYMS	GCGCTACAGCCTGAGAGATG	AGCATTTGTGGATCCCTTGAT
COL2A1	GCGACGACATAATCTGTGAAG	CTCCTTTCTGTCCCTTTGGT
IL-6	GCCACTCACCTCTTCAGAACG	GCCTCTTTGCTGCTTTCACA
MMP13	GCCATTACCAGTCTCCGAGG	CAGGCGCCAGAAGAATCTGT
ACTB	GCACAGAGCCTCGCCTT	GTTGTCGACGACGAGCG

### 2.13 miRNA-TF-mRNA regulatory network of hub OA-HRDEGs

miRNA-mRNA interactions involving hub OA-HRDEGs were predicted using experimentally validated data from miRTarBase (37) and computational predictions from TargetScan (38), accessed through the multiMiR R package. Transcription factors potentially regulating hub OA-HRDEGs were identified using Enrichr based on curated transcription factor enrichment libraries (*p* < 0.05), and miRNAs targeting the enriched transcription factors were further predicted using TargetScan. Predicted miRNA-mRNA, transcription factor-mRNA, and miRNA-transcription factor interactions were then integrated to construct the complete miRNA-TF-mRNA regulatory network, which was visualized using Cytoscape software (version 3.10.3). To improve the readability of the main figure, a simplified core regulatory network was generated from the complete network for visualization, while the complete predicted network was retained as a supplementary figure.

### 2.14 Immune-associated transcriptomic profiling

Immune-associated pathway activity and immune-cell-associated transcriptomic signals were analyzed using multiple computational approaches in R. Immune pathway activity was quantified using gene set variation analysis (GSVA) based on Hallmark and KEGG gene sets obtained from MSigDB and KEGGREST, respectively (39, 40). Differential enrichment between OA and control samples was assessed using the limma package with empirical Bayes moderation.

Immune-cell-associated transcriptomic signals were estimated using CIBERSORT with the LM22 signature matrix (41). Because cartilage is avascular and bulk transcriptomes cannot determine cellular localization, these values were interpreted as inferred immune-cell-associated deconvolution signals rather than direct measurements of infiltrating immune cells. Quantile normalization was disabled to ensure compatibility with the batch-corrected expression matrix derived from multiple microarray platforms. Group differences in immune cell proportions were evaluated using the Wilcoxon rank-sum test with false discovery rate correction. In addition, single-sample gene set enrichment analysis (ssGSEA) was used to calculate immune-cell-related and immune-function-related enrichment scores (42), which were interpreted as pathway-level transcriptomic signatures. HLA genes were identified from the integrated expression matrix based on gene symbols beginning with “HLA-”. Associations between the expression levels of hub OA-HRDEGs and immune-associated features, including immune-cell signatures, immune-function signatures, and HLA gene expression levels, were assessed using Spearman correlation analysis.

### 2.15 Statistical analysis

All statistical analyses were performed using R software (version 4.4.3) and GraphPad Prism 10.6.1. Comparisons between OA and control groups were conducted using the Wilcoxon rank-sum test, with multiple testing correction performed using the Benjamini-Hochberg method. Correlations between the expression levels of hub OA-HRDEGs and immune features were assessed using Spearman correlation analysis. Diagnostic performance of individual genes and the nomogram-based model was evaluated using ROC curve analysis, with area under the curve values calculated using the pROC package (43). Model discrimination was further quantified using Harrell’s concordance index, and calibration was assessed using bootstrap-based calibration curves. For qRT-PCR experiments, data normality was assessed using the Shapiro-Wilk test, and group comparisons were performed using Welch’s *t* test. A two-sided *p* < 0.05 or adjusted *q* < 0.05 was considered statistically significant.

## 3 Results

### 3.1 Integrated gene expression dataset after batch-effect correction

**Figure 2:**
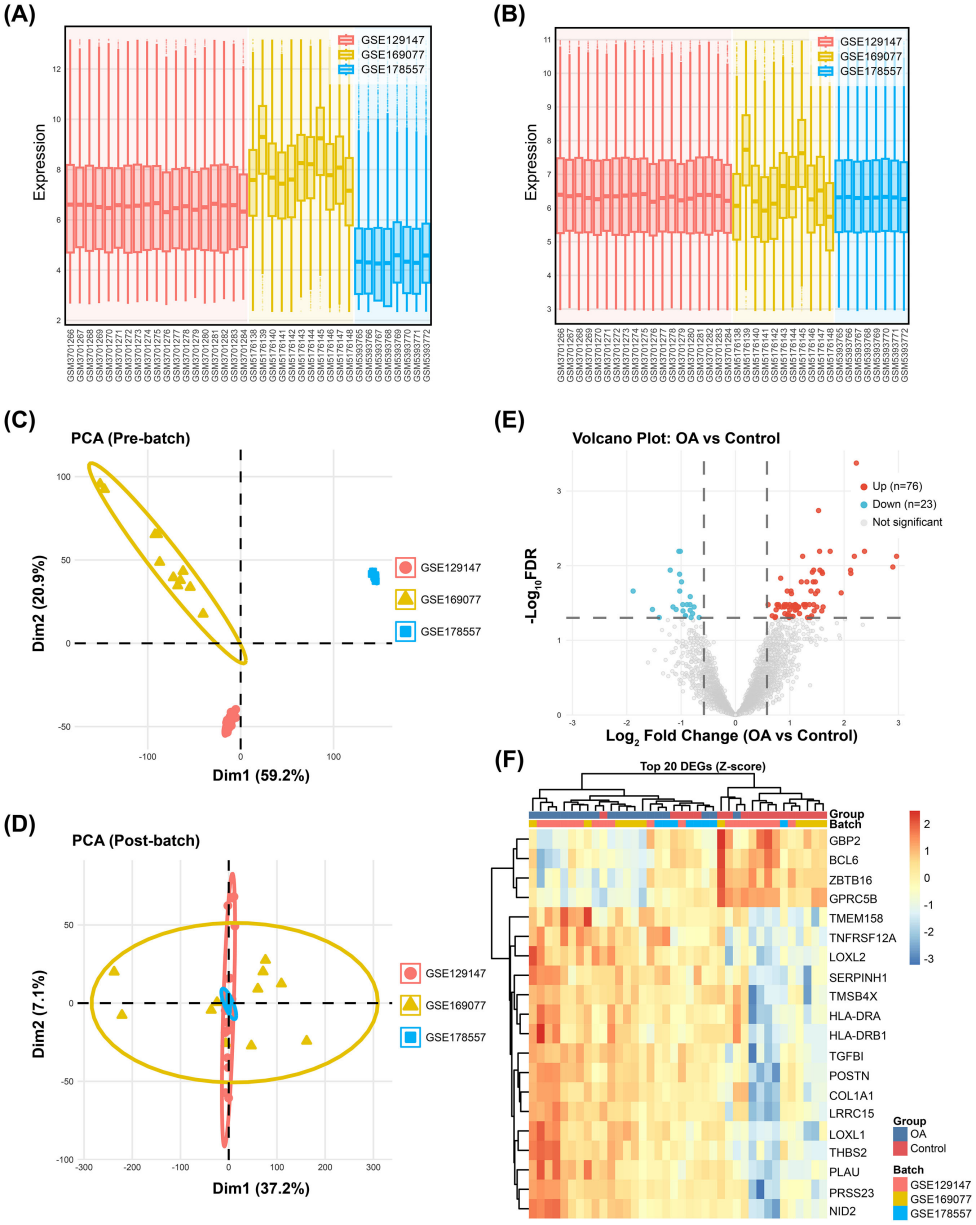
Integrated transcriptomic analysis and identification of OA-associated DEGs. **(A, B)** Boxplots of gene expression levels in the integrated training cohort before and after batch-effect correction; the horizontal axis represents samples and the vertical axis represents gene expression levels. **(C, D)** PCA plots of the integrated samples before and after batch-effect correction, showing the distribution of samples based on principal components. **(E)** Volcano plot of DEGs between OA and control cartilage; the horizontal axis represents log_2_ fold change and the vertical axis represents −log_10_(*P* value). **(F)** Heatmap of the top 20 DEGs; rows represent genes, columns represent samples, and colors indicate row-wise standardized expression levels.

After integration of the three training datasets, a batch-corrected expression matrix comprising 20 OA and 18 control cartilage samples was obtained. Because these datasets were derived from different experimental platforms and batches, substantial differences in gene expression distributions were observed prior to integration. Boxplot visualization revealed pronounced inconsistencies across samples before batch correction, indicating the presence of significant batch effects (**Figure 2A**).

To minimize technical variability and improve cross-dataset comparability, batch-effect correction was applied to the merged expression matrix using the ComBat algorithm implemented in the sva R package. Following correction, gene expression distributions across all samples became markedly more comparable, and inter-dataset variability was substantially reduced, suggesting that batch effects had been effectively mitigated (**Figure 2B**). Principal component analysis (PCA) further confirmed these findings: before batch correction, samples from different datasets were clearly separated along the first two principal components, reflecting strong batch-driven clustering (**Figure 2C**). In contrast, after batch correction, samples no longer segregated by dataset, and the biological separation between OA and control cartilage samples became more apparent (**Figure 2D**). Together, these results indicate that the integrated and batch-corrected training dataset was suitable for subsequent downstream analyses and diagnostic model construction. In addition, GSE113825 and GSE114007 datasets generated on different microarray platforms were used as independent external validation cohorts.

### 3.2 Identification and expression patterns of DEGs

A total of 99 DEGs were identified between OA cartilage samples and control cartilage samples in the batch-corrected training cohort, including 76 upregulated genes and 23 downregulated genes in OA cartilage. The complete list of OA-related DEGs was provided in **Supplementary Table 2**.

Volcano plot visualization demonstrated a clear global distribution pattern of DEGs, highlighting distinct transcriptional differences between OA and control samples (**Figure 2E**). Hierarchical clustering analysis was further conducted for the identified DEGs, and a heatmap of the top 20 most significantly differentially expressed genes revealed a pronounced separation between OA and control groups (**Figure 2F**). Several genes exhibited relatively large absolute log_2_ fold changes, indicating marked transcriptional alterations associated with OA cartilage.

Collectively, these results indicate that OA cartilage displays a distinct disease-associated transcriptional profile, providing a molecular foundation for subsequent identification of HRDEGs and downstream integrative analyses.

### 3.3 Identification of HRDEGs

A total of 63 HRDEGs were identified by overlapping the curated HRG set with the OA-associated DEGs derived from the batch-corrected training cohort. The overlap between the two gene sets was visualized using a Venn diagram (**Supplementary Figure 1**). Among these HRDEGs, 45 genes were upregulated and 18 genes were downregulated in OA cartilage. The complete list of HRDEGs was provided in **Supplementary Table 3**.

### 3.4 Construction of PPI networks

**Figure 3:**
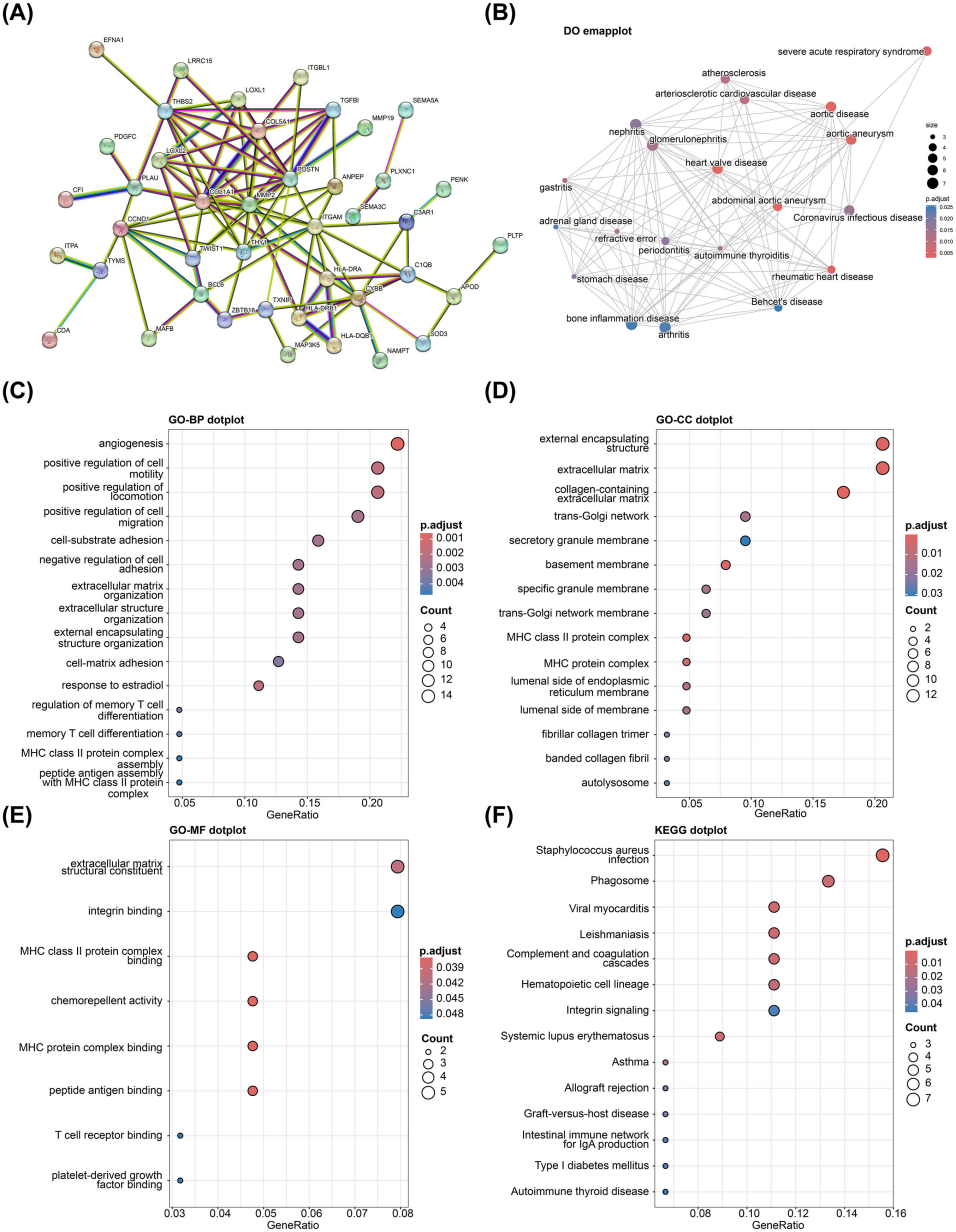
PPI network and functional enrichment analysis of HRDEGs. **(A)** The PPI network of HRDEGs; nodes represent genes and edges represent protein–protein interactions. **(B)** DO emapplot displays the top 20 enriched disease terms; nodes represent enriched disease terms and edges indicate shared genes between terms. **(C–E)** GO enrichment dot plots for BP, CC, and MF of HRDEGs; the horizontal axis represents gene ratio, the vertical axis represents GO terms, dot size indicates gene counts, and dot color represents the significance level. **(F)** KEGG pathway enrichment dot plot of HRDEGs; the horizontal axis represents gene ratio, the vertical axis represents KEGG pathways, dot size indicates gene counts, and dot color represents the significance level.

To characterize potential functional connectivity among the HRDEGs at the protein level, a PPI network was generated using STRING with a minimum required interaction score of 0.4. The resulting network summarizes putative interaction relationships among the identified HRDEGs (**Figure 3A**), and the corresponding interaction details were provided in **Supplementary Table 4**.

### 3.5 Functional enrichment analysis of HRDEGs

Functional enrichment analyses revealed that the identified HRDEGs were enriched in annotation terms related to extracellular remodeling, inflammatory signaling, antigen presentation, and several immune/infection-associated pathways. DO enrichment analysis indicated significant associations with cardiovascular and inflammation-related disease categories, with the top enriched terms including rheumatic heart disease, heart valve disease, aortic aneurysm, abdominal aortic aneurysm, and aortic disease, as shown in the top 20 DO terms displayed in the emapplot (**Figure 3B**).

GO enrichment analysis further demonstrated that HRDEGs were significantly enriched in biological processes related to tissue remodeling and cellular dynamics, including angiogenesis, response to estradiol, positive regulation of cell motility, positive regulation of locomotion, and cell-substrate adhesion (**Figure 3C**). In the cellular component category, the most significantly enriched terms were external encapsulating structure, extracellular matrix, and collagen-containing extracellular matrix, indicating a predominant association with extracellular structural components (**Figure 3D**). Molecular function analysis revealed enrichment in immune-related activities, including MHC class II protein complex binding, chemorepellent activity, and MHC protein complex binding (**Figure 3E**).

Consistently, KEGG pathway enrichment analysis showed that HRDEGs were significantly involved in immune- and inflammation-associated pathways, such as Staphylococcus aureus infection, viral myocarditis, leishmaniasis, complement and coagulation cascades, and systemic lupus erythematosus (**Figure 3F**). Detailed results of the DO, GO, and KEGG enrichment analyses were provided in **Supplementary Tables 5**‐**9**.

### 3.6 WGCNA-based identification of OA-associated key modules and functions

WGCNA was applied to identify gene modules associated with OA in the batch-corrected training cohort. Following quality control, three samples showing marked deviation from the main sample cluster were excluded, and the remaining 35 samples and 11,836 genes were retained for network construction (**Supplementary Figure 2A**). The sample dendrogram and trait heatmap demonstrated clear separation between OA and control samples, with no additional outliers detected (**Supplementary Figure 2B**).

**Figure 4:**
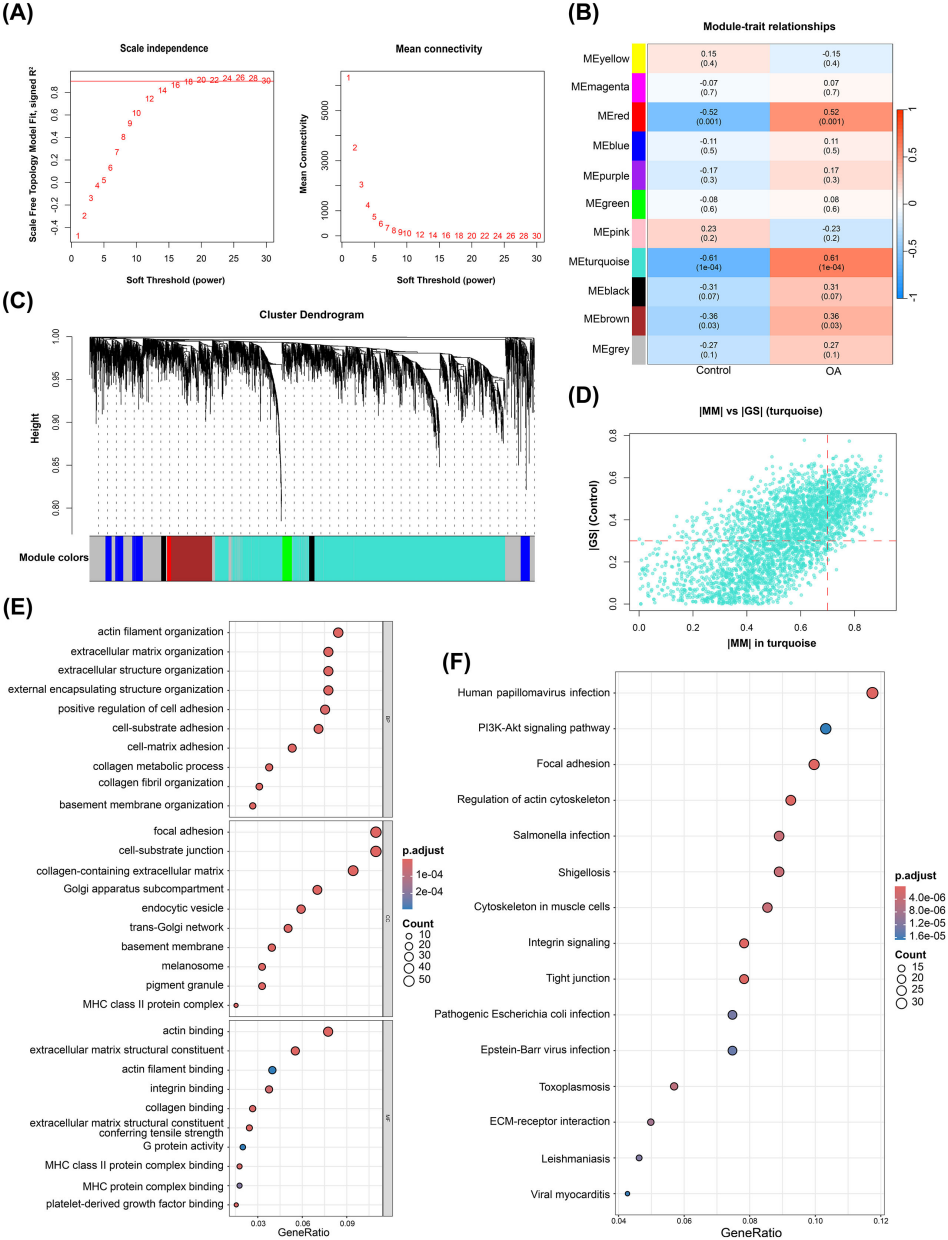
WGCNA identifies OA-associated gene modules and functions. **(A)** Scale-free topology model fit and mean connectivity plots; the horizontal axis represents the soft-thresholding power, and the vertical axis represents the scale-free topology model fit index or mean connectivity. **(B)** Module-trait relationship heatmap; colors represent the correlation between module eigengenes and OA status. **(C)** Gene clustering dendrogram based on topological overlap, with different colors indicating distinct co-expression modules. **(D)** Scatter plot of MM versus GS for genes in the turquoise module. **(E)** GO enrichment dot plot for BP, CC, and MF terms of genes in the turquoise module; the horizontal axis represents gene ratio and the vertical axis represents GO terms. **(F)** KEGG pathway enrichment dot plot for genes in the turquoise module; the horizontal axis represents gene ratio and the vertical axis represents KEGG pathways.

An appropriate soft-thresholding power was selected to achieve approximate scale-free topology. The soft-thresholding analysis supported β = 16, at which the scale-free topology fit index reached approximately 0.90 while maintaining acceptable mean connectivity, and a signed weighted gene co-expression network was constructed (**Figure 4A**). Using dynamic tree cutting and module merging, a total of 11 distinct co-expression modules were identified from the gene dendrogram and module color assignment (**Figure 4C**; **Supplementary Figures 2C, D**). Among these modules, the turquoise module, comprising 3,161 genes, exhibited the strongest positive correlation with the OA phenotype (cor = 0.61, p < 0.001) and was therefore selected as the key OA-associated module according to module–trait relationships (**Figure 4B**). The top 30 genes ranked by intramodular connectivity (kWithin) within the turquoise module were shown in **Supplementary Figure 2E**.

Within the turquoise module, GS and MM were calculated, and hub genes were identified using the criteria |MM| ≥ 0.70 and |GS| ≥ 0.30 (**Figure 4D**). The resulting hub genes were listed in **Supplementary Table 10**. Functional enrichment analysis of these genes revealed predominant associations with extracellular matrix remodeling and cell adhesion-related biological processes. GO biological process analysis showed significant enrichment in extracellular matrix organization, extracellular structure organization, external encapsulating structure organization, actin filament organization, and basement membrane organization (**Figure 4E**). Consistently, KEGG pathway analysis indicated enrichment in focal adhesion, regulation of actin cytoskeleton, tight junction, human papillomavirus infection, and Salmonella infection pathways, highlighting functional links to cell-matrix interactions, cytoskeletal dynamics, and immune- or infection-related processes (**Figure 4F**). Detailed GO and KEGG enrichment results were provided in **Supplementary Tables 11** and **12**.

### 3.7 Identification of OA-HRDEGs

**Figure 5:**
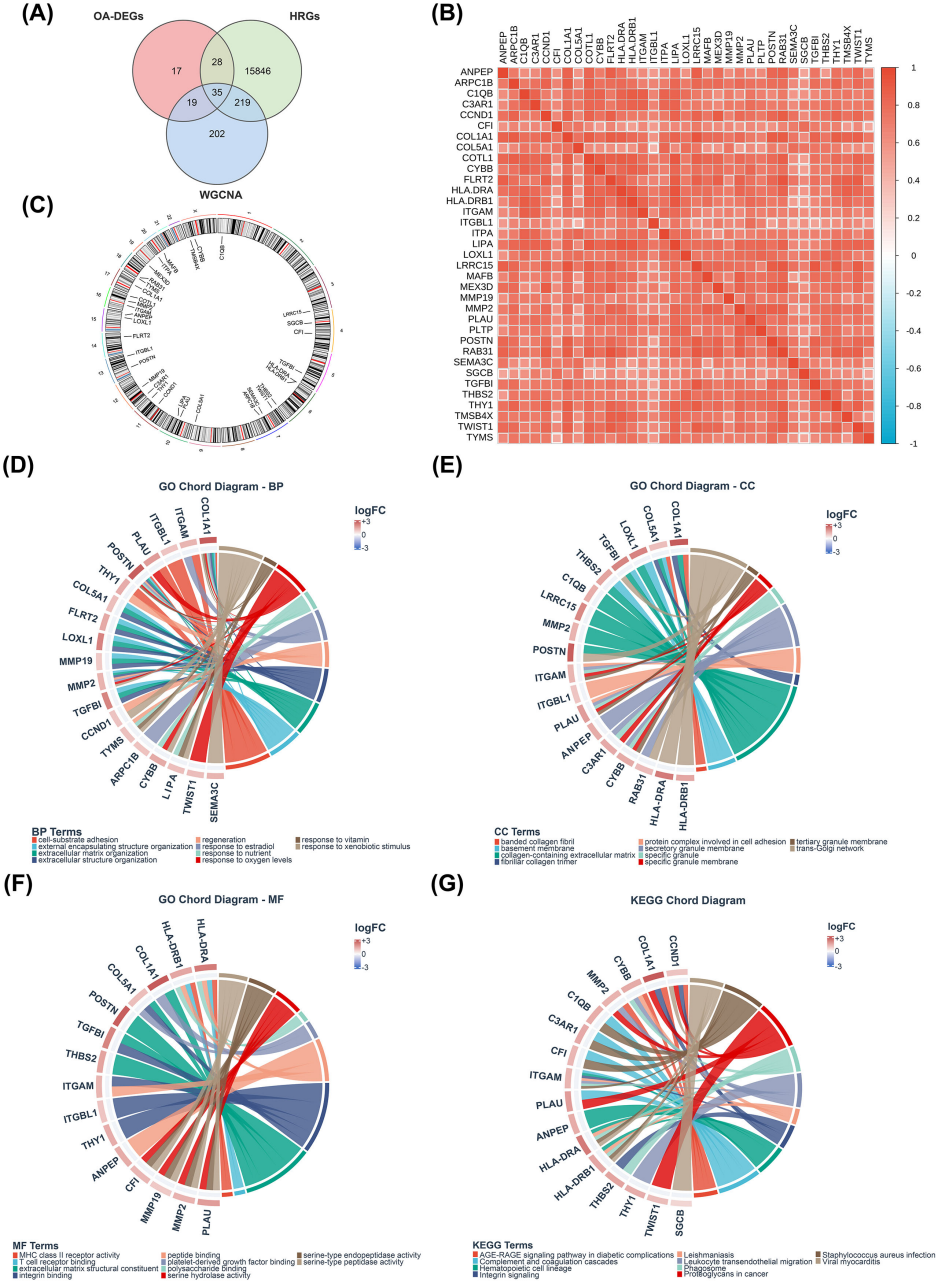
Identification and functional characterization of OA-HRDEGs. **(A)** Venn diagram showing the overlap among OA-DEGs, HRGs, and WGCNA-derived hub genes. **(B)** Correlation heatmap of OA-HRDEGs based on pairwise expression correlations. **(C)** Chromosomal distribution of OA-HRDEGs, showing the genomic locations of the genes. **(D–F)** GO chord diagrams showing the associations between OA-HRDEGs and enriched BP, CC, and MF terms; ribbons indicate gene–term relationships and colors represent gene expression levels. **(G)** KEGG chord diagram showing the associations between OA-HRDEGs and enriched pathways; ribbons indicate gene–pathway relationships.

A total of 35 OA-HRDEGs were defined by intersecting the OA-associated DEGs, the OA-associated hub genes identified by WGCNA, and the HRDEGs. The overlap among the three gene sets was illustrated in a Venn diagram (**Figure 5A**), and the complete list of OA-HRDEGs was provided in **Supplementary Table 13**.

### 3.8 Correlation, genomic distribution, and functional analysis of OA-HRDEGs

Pairwise Pearson correlation analysis indicated that the majority of OA-HRDEGs were positively correlated with each other, suggesting coordinated expression patterns within this gene set (**Figure 5B**). The correlation matrix was provided in **Supplementary Table 14**. Chromosomal localization analysis showed that OA-HRDEGs were distributed across multiple autosomes and the X chromosome, without evidence of prominent chromosomal clustering (**Figure 5C**; **Supplementary Table 15**).

Functional enrichment analysis further characterized the biological features of OA-HRDEGs. GO biological process enrichment highlighted extracellular matrix organization, extracellular structure organization, and external encapsulating structure organization (**Figure 5D**). In the cellular component category, OA-HRDEGs were enriched in collagen-containing extracellular matrix, secretory granule membrane, and specific granule membrane (**Figure 5E**). Molecular function analysis showed enrichment in integrin binding, extracellular matrix structural constituent activity, and MHC class II receptor activity (**Figure 5F**). Consistently, KEGG pathway enrichment analysis indicated involvement in immune- and infection-related pathways, including Staphylococcus aureus infection, complement and coagulation cascades, and viral myocarditis (**Figure 5G**). Detailed GO and KEGG enrichment results were provided in **Supplementary Tables 16** and **17**.

### 3.9 Identification of hub OA-HRDEGs

**Figure 6:**
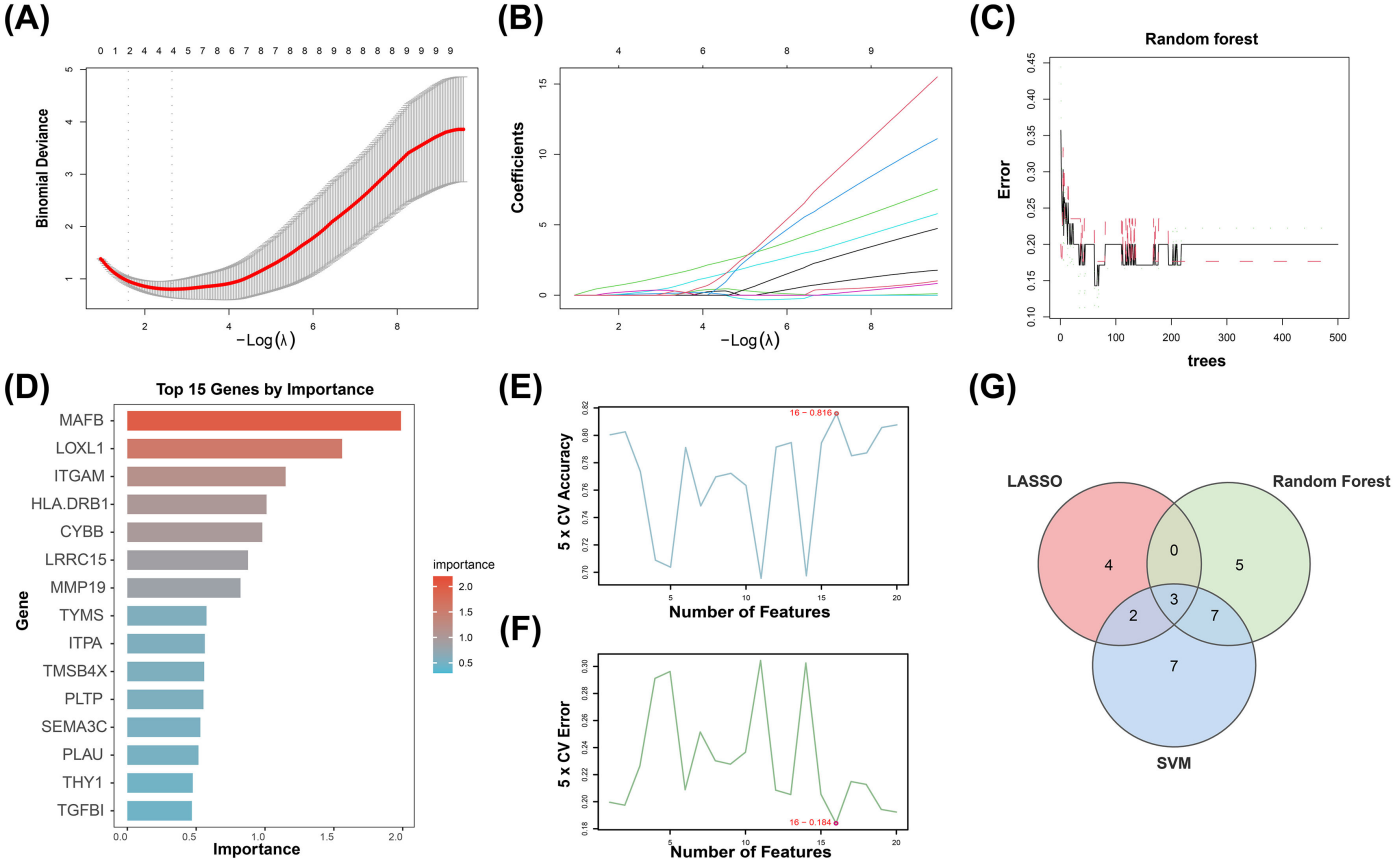
Machine-learning-based identification of hub OA-HRDEGs. **(A, B)** LASSO regression analysis showing coefficient profiles and selection of features under the optimal penalty parameter. **(C, D)** Random forest analysis showing model error and feature importance ranking. **(E, F)** SVM-RFE analysis showing classification accuracy and error rates with different feature numbers. **(G)** Venn diagram showing the overlap of candidate genes identified by LASSO, SVM-RFE, and random forest.

Using three complementary machine-learning approaches (LASSO, SVM-RFE, and random forest), robust diagnostic features were identified from the OA-HRDEGs. LASSO regression yielded candidate genes with non-zero coefficients under the optimal penalty parameter (**Figures 6A, B**). SVM-RFE selected an optimal feature subset by recursively eliminating variables to minimize classification error (**Figures 6E, F**). Random forest analysis further ranked candidate genes based on feature importance (**Figures 6C, D**). Intersecting the outputs of these three methods identified three consensus hub OA-HRDEGs—LOXL1, THY1, and TYMS (**Figure 6G**). Detailed results from each feature-selection method were provided in **Supplementary Table 18**.

### 3.10 Construction of a Hub OA-HRDEGs-based classification model

**Figure 7:**
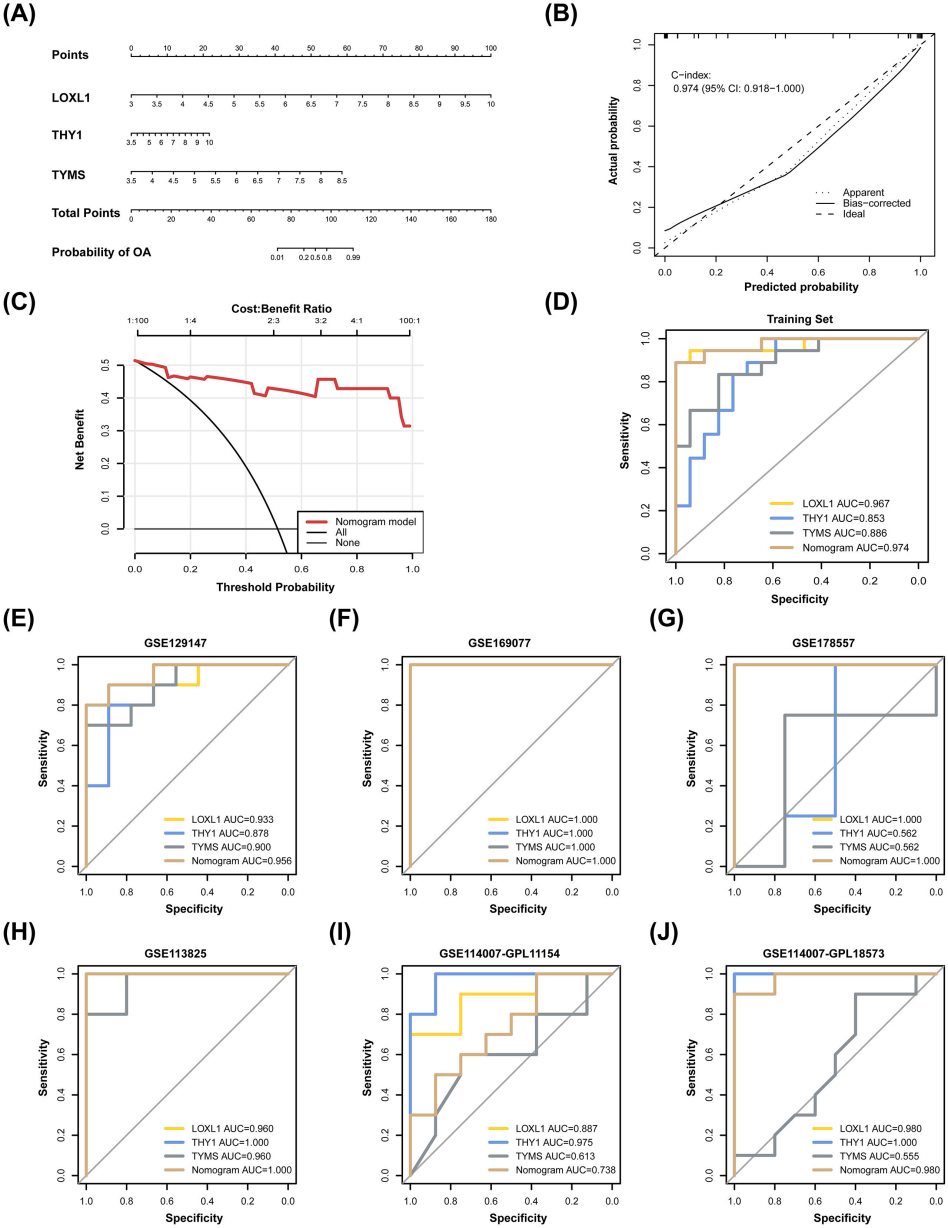
Construction and validation of the hub OA-HRDEGs-based OA classification model. **(A)** Nomogram constructed based on the expression levels of LOXL1, THY1, and TYMS for OA classification. **(B)** Calibration curve of the nomogram; the horizontal axis represents predicted probability and the vertical axis represents observed probability. **(C)** Decision curve analysis of the nomogram in the training cohort, showing the net benefit across a range of threshold probabilities. **(D)** ROC curves of individual hub genes and the nomogram in the training cohort; the horizontal axis represents specificity and the vertical axis represents sensitivity. **(E–G)** ROC curves in each training dataset. **(H–J)** ROC curves in independent validation datasets.

A multivariable logistic regression-based OA classification model incorporating LOXL1, THY1, and TYMS was constructed and visualized as a nomogram (**Figure 7A**). Calibration analysis demonstrated agreement between predicted probabilities and observed outcomes, and the nomogram also showed strong discriminative performance, with a C-index of 0.974 (95% CI: 0.918–1.000) (**Figure 7B**). Decision curve analysis showed that the nomogram provided a positive net benefit across a range of threshold probabilities in the training cohort (**Figure 7C**). Hub gene expression values and the corresponding nomogram-derived classification scores were provided in **Supplementary Table 19**.

### 3.11 Expression patterns and discriminative value of hub OA-HRDEGs

Consistent with their selection as hub features, LOXL1, THY1, and TYMS showed higher expression in OA cartilage than in controls across the training and independent validation cohorts (**Supplementary Figures 3A–D**). ROC curve analysis demonstrated that all three hub genes and the nomogram-based model exhibited good diagnostic performance in the training cohort (**Figure 7D**). The nomogram demonstrated the highest discriminative ability (AUC = 0.974), outperforming all individual hub genes, among which LOXL1 showed the strongest single-gene discrimination (AUC = 0.967), followed by TYMS (AUC = 0.886) and THY1 (AUC = 0.853) (**Figure 7D**).

ROC analyses performed within each training dataset (GSE129147, GSE169077, and GSE178557) were shown in **Figures 7E–G**. In the independent external validation cohorts, the diagnostic performance of each hub gene and the nomogram-based score was further evaluated by AUC values with 95% confidence intervals. In GSE113825, the AUC values were 0.960 for LOXL1 (95% CI: 0.849–1.000), 1.000 for THY1 (95% CI: 1.000–1.000), 0.960 for TYMS (95% CI: 0.849–1.000), and 1.000 for the nomogram-based score (95% CI: 1.000–1.000) (**Figure 7H**). In GSE114007-GPL11154, the corresponding AUC values were 0.887 for LOXL1 (95% CI: 0.732–1.000), 0.975 for THY1 (95% CI: 0.916–1.000), 0.613 for TYMS (95% CI: 0.334–0.891), and 0.738 for the nomogram-based score (95% CI: 0.498–0.977) (**Figure 7I**). In GSE114007-GPL18573, the AUC values were 0.980 for LOXL1 (95% CI: 0.933–1.000), 1.000 for THY1 (95% CI: 1.000–1.000), 0.555 for TYMS (95% CI: 0.284–0.826), and 0.980 for the nomogram-based score (95% CI: 0.933–1.000) (**Figure 7J**). These results suggest that the three-gene signature and nomogram-based score retained potential tissue-level classification value in external validation cohorts, although performance varied across platforms and further validation in larger prospective datasets is needed.

### 3.12 qRT-PCR

**Figure 8:**
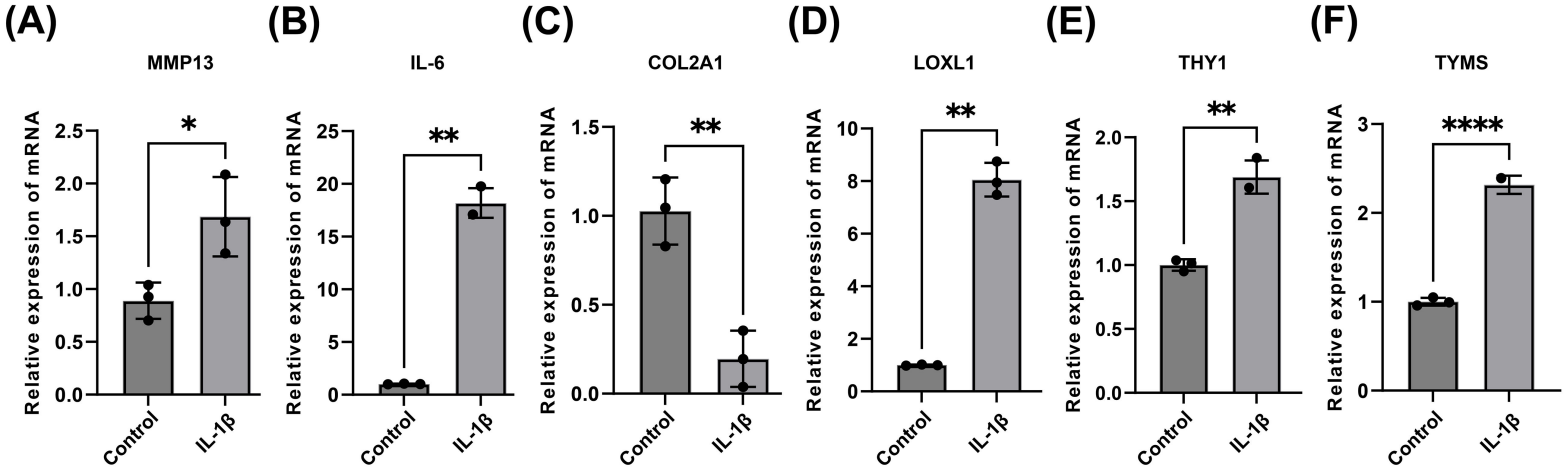
qRT-PCR validation of hub OA-HRDEGs in an IL-1β-stimulated C28/I2 chondrocyte model. Relative mRNA expression of MMP13 **(A)**, IL-6 **(B)**, COL2A1 **(C)**, LOXL1 **(D)**, THY1 **(E)**, and TYMS **(F)** in untreated control and IL-1β-stimulated C28/I2 chondrocytes. Data are presented as mean ± SD from three independent biological replicates. Statistical significance is indicated as *p < 0.05, **p < 0.01, and ****p < 0.0001.

To provide preliminary experimental support for the inflammatory responsiveness of the hub genes, qRT-PCR was performed in an IL-1β-stimulated inflammatory chondrocyte model. Compared with untreated controls, IL-1β stimulation significantly increased the mRNA levels of MMP13 and IL-6 and decreased COL2A1 expression, consistent with an inflammatory and catabolic shift in chondrocytes (**Figures 8A–C**).

In line with the direction observed in datasets, the mRNA expression levels of the three hub OA-HRDEGs were significantly upregulated following IL-1β stimulation, with LOXL1 showing the largest change among the three genes (**Figures 8D–F**). These results suggest that the three hub genes can be induced under inflammatory chondrocyte stress, supporting but not proving their disease-associated relevance. Raw Ct values and 2^−ΔΔCt^ calculations were provided in **Supplementary Table 20**.

### 3.13 miRNA-TF-mRNA regulatory network

**Figure 9:**
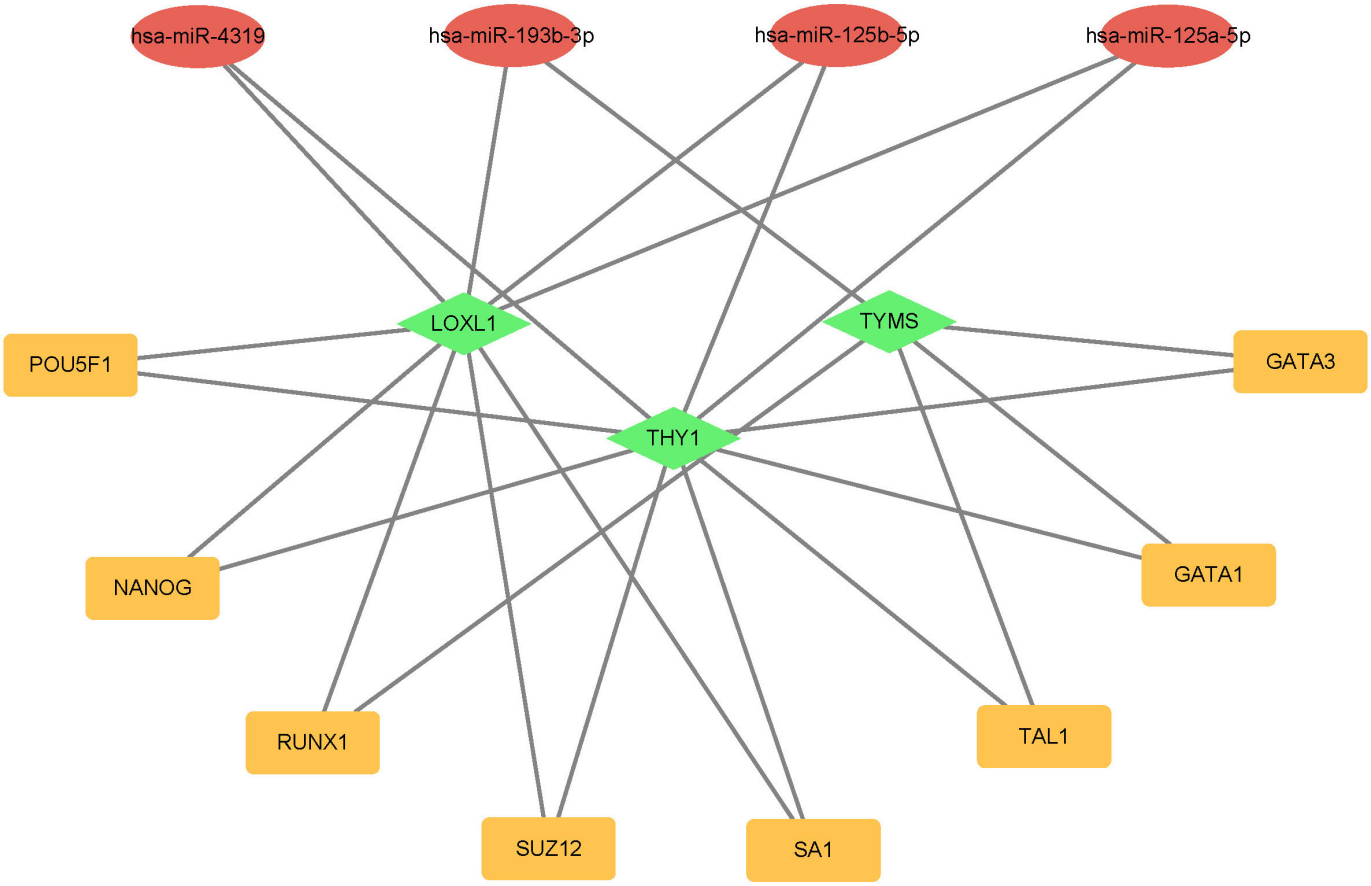
Core miRNA–TF–mRNA regulatory network of hub OA-HRDEGs. The main figure displays a simplified core network of key regulatory nodes associated with LOXL1, THY1, and TYMS. Red oval nodes represent miRNAs, yellow rectangular nodes represent transcription factors, and green diamond nodes represent hub OA-HRDEGs. Gray lines indicate predicted regulatory interactions.

To generate hypotheses regarding potential upstream regulatory relationships of the hub OA-HRDEGs, miRNA- and transcription factor-based regulatory relationships targeting LOXL1, THY1, and TYMS were integrated to construct a miRNA-TF-mRNA network. The resulting network comprised 55 miRNAs, 88 transcription factors, and three hub genes, with a total of 205 putative regulatory interactions. For clarity, **Figure 9** displays a simplified core network including only regulators connected to at least two hub genes. The complete regulatory network was provided in **Supplementary Figure 4**, and the complete set of regulatory relationships was provided in **Supplementary Table 21**.

### 3.14 Immune-associated transcriptomic features of OA cartilage

**Figure 10:**
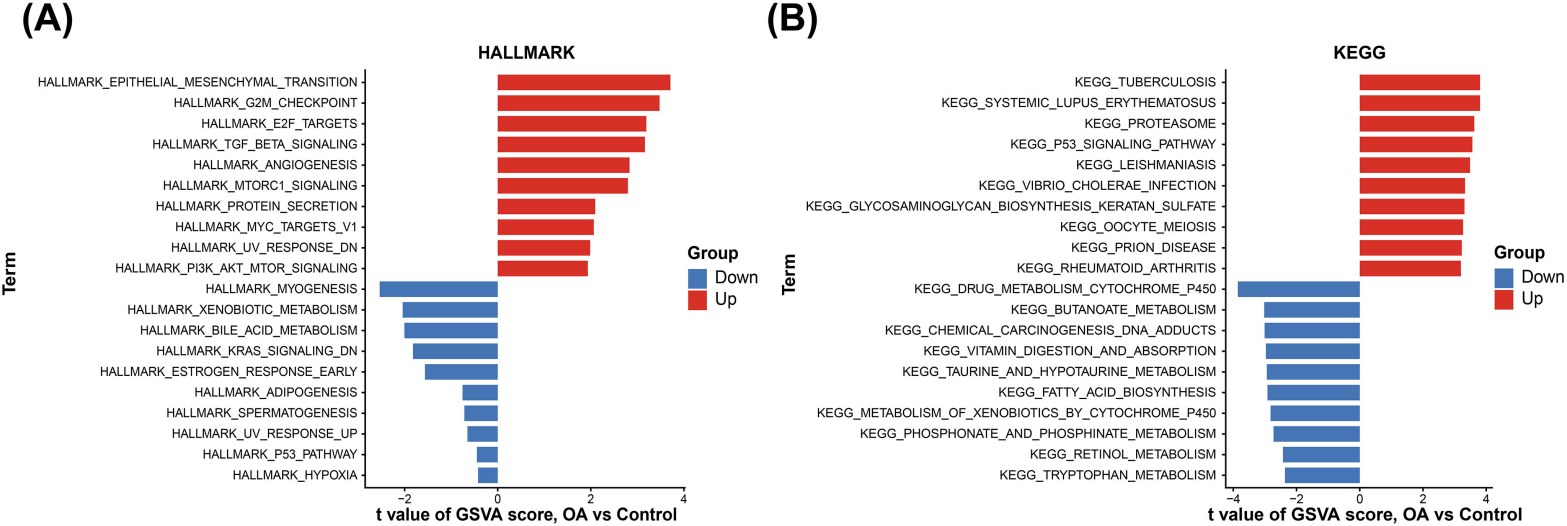
GSVA-based pathway analysis of OA and control cartilage. **(A)** Bar plot showing GSVA results for Hallmark gene sets in OA and control samples. **(B)** Bar plot showing GSVA results for KEGG pathways in OA and control samples. Bar length represents the t value of GSVA scores between groups.

To delineate the immunological characteristics of OA cartilage, we integrated GSVA-based pathway analysis, immune-cell-associated deconvolution and ssGSEA-derived immune signature profiling. GSVA indicated that OA samples were enriched for gene signatures related to cell-cycle progression, stromal/extracellular matrix remodeling, and immune-associated signaling, including the Hallmark epithelial-mesenchymal transition signature, G2/M checkpoint, E2F targets, TGF-β signaling, angiogenesis, and mTORC1 signaling. In contrast, signatures related to metabolic and detoxification processes—such as xenobiotic metabolism, bile acid metabolism, and cytochrome P450-associated pathways—were suppressed in OA samples (**Figures 10A, B**). Complete GSVA results for Hallmark and KEGG pathways were provided in **Supplementary Tables 22** and **23**.

**Figure 11:**
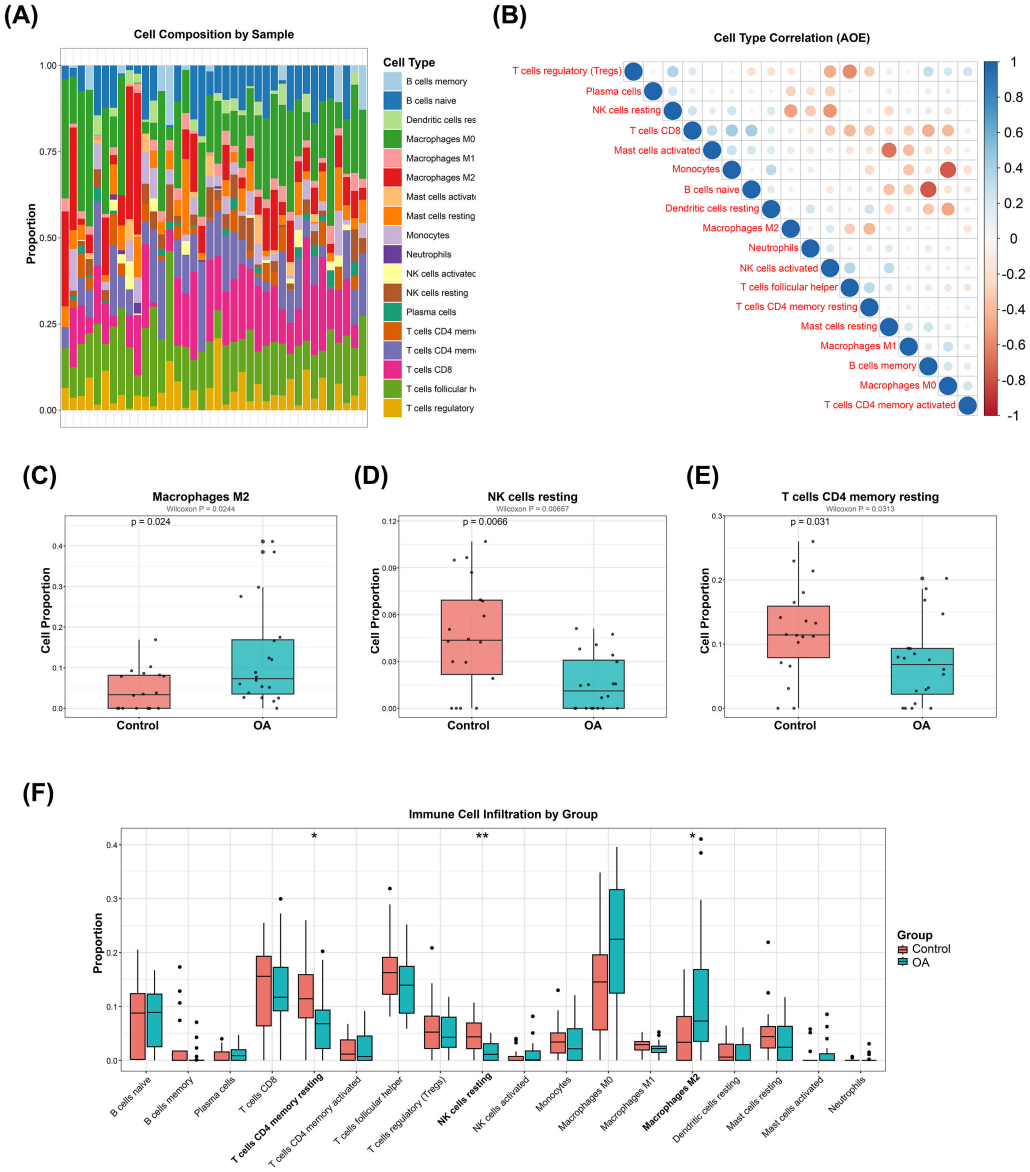
CIBERSORT-based immune-cell-associated deconvolution analysis. **(A)** Stacked bar plot showing the inferred relative deconvolution signals of immune cell types in each sample. **(B)** Correlation heatmap showing pairwise correlations among inferred immune cell types. **(C–E)** Boxplots showing differences in the proportions of macrophages M2, resting NK cells, and resting CD4 memory T cells between OA and control groups. **(F)** Boxplot showing the distribution of immune cell proportions between OA and control groups. Statistical significance is indicated as *p < 0.05 and **p < 0.01.

CIBERSORT analysis revealed marked inter-sample heterogeneity in inferred immune-cell composition while demonstrating systematic differences between OA and control groups (**Figure 11A**; **Supplementary Table 24**). CIBERSORT deconvolution suggested increased macrophage M2-associated signals in OA cartilage samples accompanied by lower resting NK-cell- and resting CD4 memory T-cell-associated deconvolution signals, as supported by Wilcoxon rank-sum testing (**Figures 11C–F**; **Supplementary Table 25**). Most other immune-cell subsets showed comparable distributions between groups. Correlation analysis of CIBERSORT-inferred immune cells suggested coordinated relationships among immune populations (**Figure 11B**), with macrophage subsets showing close associations with multiple T-cell populations, consistent with a coordinated immune-associated transcriptomic pattern rather than isolated changes.

**Figure 12:**
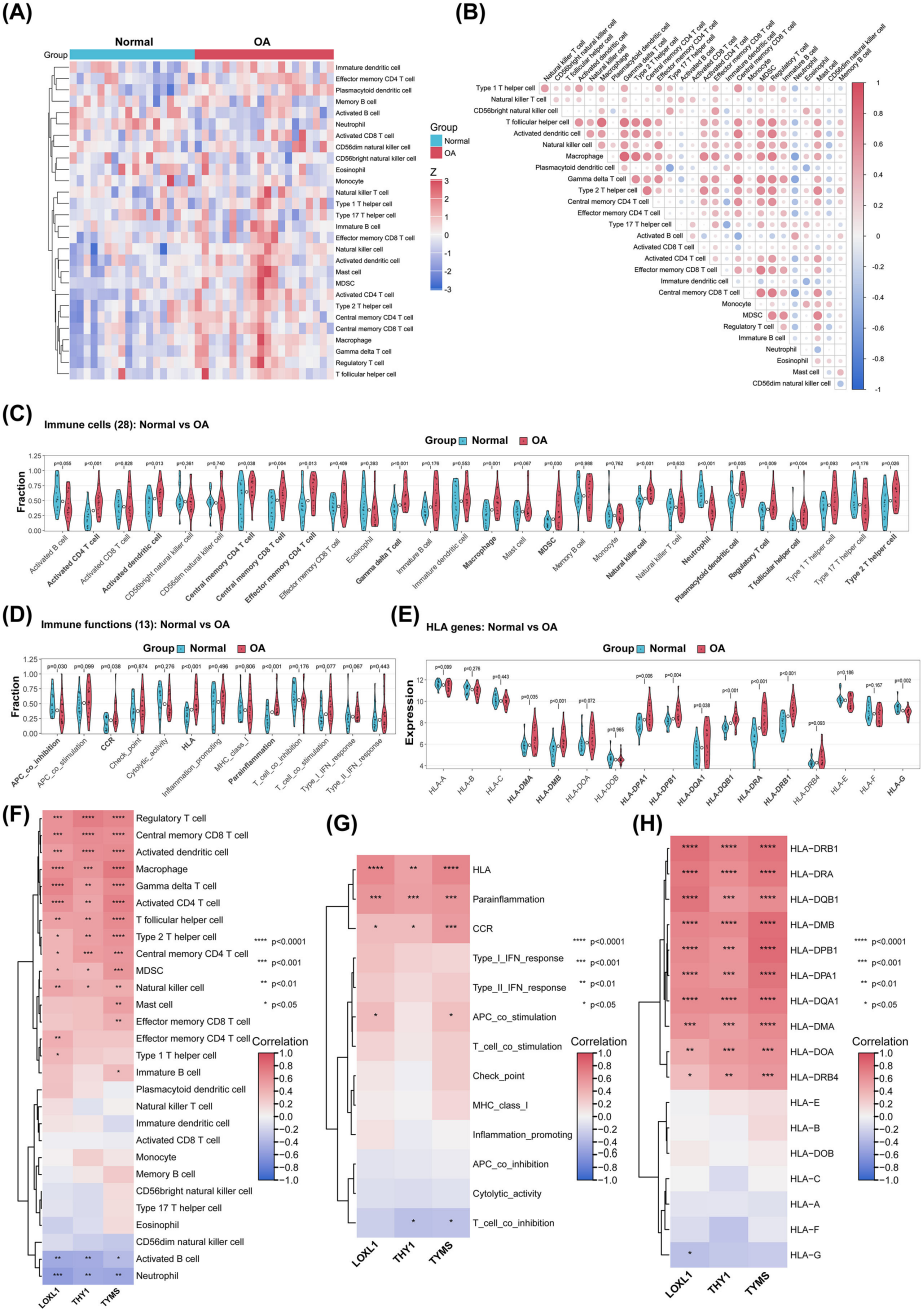
ssGSEA-based immune-associated signature profiling and correlation analysis. **(A)** Heatmap showing ssGSEA enrichment scores of immune-cell-related signatures in OA and control samples. **(B)** Correlation heatmap of immune cell signatures based on ssGSEA scores. **(C)** Violin plots showing differences in ssGSEA scores of immune cell signatures between OA and control groups. **(D)** Violin plots showing differences in ssGSEA scores of immune-related functions between OA and control groups. **(E)** Violin plots showing expression levels of HLA genes in OA and control samples. **(F)** Correlation heatmap showing associations between hub gene expression and immune cell signatures. **(G)** Correlation heatmap showing associations between hub gene expression and immune-related functions. **(H)** Correlation heatmap between hub OA-HRDEGs and HLA genes. Colors indicate correlation coefficients, and asterisks indicate statistical significance. Statistical significance is indicated as *p < 0.05, **p < 0.01, ***p < 0.001, and ****p < 0.0001.

These major deconvolution trends—particularly the macrophage-skewed signal and altered T/NK-cell-related features—were directionally supported by ssGSEA. OA samples exhibited higher ssGSEA enrichment scores for activated immune-cell-related signatures, including activated CD4 T cells, activated CD8 T cells, activated dendritic cells, macrophages, regulatory T cells, natural killer T cells, and effector memory T-cell subsets (**Figures 12A, C**), whereas central memory CD4 and central memory CD8 T-cell signatures were reduced in OA compared with controls (**Figure 12C**). Correlation analysis based on ssGSEA scores further demonstrated coordinated relationships among immune signatures, with macrophage- and T-cell-related signatures forming closely associated transcriptomic clusters (**Figure 12B**). Full ssGSEA results for immune cell signatures were available in **Supplementary Table 26**.

At the immune-function level, ssGSEA indicated that OA samples showed increased enrichment of antigen presentation- and immune regulation-related functions, including HLA, parainflammation, CCR signaling, and APC co-inhibition (**Figure 12D**). Consistent with these functional changes, several HLA class II genes (HLA-DRA, HLA-DRB1, HLA-DQB1, HLA-DMB, and HLA-DPB1) were upregulated in OA samples (**Figure 12E**). Full ssGSEA results for immune-related functions were available in **Supplementary Table 27**.

Furthermore, correlation analyses showed that expression levels of the hub genes LOXL1, THY1, and TYMS were positively associated with macrophage and activated T-cell signatures, regulatory T-cell signatures, and antigen presentation-related immune functions, while showing negative associations with neutrophil and activated B-cell signatures (**Figures 12F, G**). In addition, the three hub genes were positively correlated with multiple HLA class II genes, including HLA-DRA, HLA-DRB1, HLA-DQB1, HLA-DMB, HLA-DPA1, and HLA-DPB1 (**Figure 12H**; **Supplementary Table 28**). Together, these results support an immune-associated OA cartilage microenvironment characterized by macrophage- and activated T-cell-associated signals with enhanced antigen presentation and immune regulatory features.

## 4 Discussion

In this study, we integrated multiple GEO cartilage transcriptomic datasets to identify annotation-derived Hb-associated immunotranscriptomic signatures in OA and to examine their relationships with immune-associated transcriptomic features in OA cartilage. These genes were consistently upregulated across training and validation cohorts and were preliminarily supported by qRT-PCR in an IL-1β-stimulated inflammatory chondrocyte model. Immune-associated transcriptomic profiling further linked this three-gene signature to macrophage-/T-cell-related signals and antigen-presentation-related activity, suggesting that it may reflect immune-associated remodeling states in late-stage OA cartilage.

Here, “Hb-associated” should be interpreted as an operational annotation term rather than as a direct reference to canonical hemoglobin-subunit genes. The HRG set was derived from the GeneCards keyword “Hemoglobin” and therefore represents a broad Hb-associated annotation space. This strategy was chosen to maximize sensitivity for detecting genes directly or indirectly linked to oxygen handling, hypoxic adaptation, oxidative stress, erythroid-associated biology, and immune-related responses. However, this broad definition may introduce annotation noise. Importantly, pipeline-level tracking of representative classical globin and heme-related genes showed that genes such as HBA1, HBA2, HBB, HBD, and ALAS2 were not retained among the final OA-HRDEGs or the three hub genes (**Supplementary Table 29**). Therefore, LOXL1, THY1, and TYMS should be interpreted as annotation-derived Hb-associated biomarkers rather than classical hemoglobin genes.

Articular cartilage is an avascular tissue with a physiologically hypoxic microenvironment, and chondrocytes must adapt to sustained low oxygen tension and fluctuating metabolic stress (44, 45). Recent transcriptomic studies suggest that Hb-associated gene programs can be detected in specific chondrocyte subpopulations and may reflect stress-adaptive states relevant to cartilage biology (46, 47). In line with this concept, the enrichment patterns observed for our HRDEGs and OA-HRDEGs—encompassing inflammatory/immune pathways, cellular stress responses, and extracellular matrix (ECM) remodeling—support the notion that Hb-associated transcriptional signals in OA cartilage are embedded within broader inflammatory-remodeling programs.

The absence of representative classical globin and heme-related genes from the final candidates may reflect their low or inconsistent detectability in cartilage microarray profiles and the cross-platform filtering strategy, which preferentially retained robust remodeling- and immune-associated transcriptional programs within the Hb-associated annotation space (48, 49). Although perioperative blood contamination or adjacent-tissue admixture cannot be fully excluded in public cartilage datasets (50), the lack of canonical globin transcripts among the final candidates argues against simple erythrocyte-driven contamination as the dominant explanation.

Biologically, the three hub genes converge on processes plausibly relevant to OA progression. LOXL1 is linked to ECM organization and collagen/elastin cross-linking (51), aligning with our WGCNA results in which the key OA-associated module was enriched for extracellular matrix organization, cell-matrix adhesion, focal adhesion, and cytoskeletal regulation. Such ECM remodeling programs can shape tissue mechanics and modify cell-matrix signaling, potentially influencing immune-associated transcriptional activation in diseased cartilage. THY1 (CD90) is widely used as a stromal activation and adhesion-associated marker and may reflect an activated cellular phenotype within the OA cartilage milieu (52–54). TYMS, a key enzyme in thymidylate biosynthesis, may reflect proliferative or stress-associated transcriptional activity (55), consistent with the enrichment of cell-cycle-related signatures (e.g., G2/M checkpoint and E2F targets) observed in OA samples. Together, these genes appear to integrate ECM remodeling, cellular activation, and stress/cell-cycle-associated transcription into a measurable Hb-associated OA signature.

Because articular cartilage is avascular and predominantly composed of chondrocytes, immune deconvolution results from bulk cartilage transcriptomes should be interpreted cautiously. The macrophage- and T-cell-associated signals detected in this study may reflect several non-mutually exclusive sources, including low-level immune/stromal cell admixture, adjacent synovial or subchondral tissue contamination, residual blood-derived signals, and inflammatory co-regulation of chondrocyte transcriptional programs. Therefore, our findings should not be interpreted as direct quantification of immune-cell infiltration within the cartilage layer. Instead, they support the presence of immune-associated transcriptomic activity accompanying OA-related tissue remodeling.

From an immunological perspective, our multi-method profiling supports the presence of immune-associated transcriptional activation in late-stage OA cartilage. CIBERSORT suggested increased inferred M2 macrophage signals with reduced resting NK-cell and resting CD4 memory T-cell signals, while ssGSEA showed elevated signatures of activated immune subsets (including activated T-cell and dendritic-cell signatures) and increased immune functions related to antigen presentation and immune regulation (e.g., HLA-related activity, parainflammation, CCR signaling, and APC co-inhibition) (**Figures 11**, **12**). The concordant upregulation of multiple HLA class II genes, together with their positive correlations with LOXL1, THY1, and TYMS, further supports an association between the hub-gene signature and antigen presentation-related transcriptomic activity. These findings should therefore be interpreted as coordinated immune-associated transcriptomic features rather than direct immune-cell quantification (56).

The positive associations between hub gene expression (LOXL1, THY1, TYMS) and macrophage-/activated T-cell-related signatures and antigen presentation-related functions suggest that this three-gene axis may serve as a transcriptomic readout of immune-associated remodeling states in OA cartilage. Although correlation does not establish causality, the observed relationships are consistent with a bidirectional interplay in which ECM remodeling and altered cell-matrix signaling can modulate immune activation, while immune-derived cytokines and inflammatory mediators may reciprocally amplify chondrocyte catabolism, stromal activation, and structural remodeling (57, 58). Thus, our data provide an immunotranscriptomic framework linking Hb-associated annotation space, ECM remodeling programs, and immune-associated transcriptomic signatures in OA cartilage.

Nevertheless, the present correlation analyses cannot determine whether LOXL1, THY1, and TYMS promote immune activation or are induced as a consequence of inflammatory stimulation. A plausible interpretation is that these genes and immune-associated signatures are co-regulated by inflammatory mediators and disease-associated microenvironmental stressors, including IL-1β, TNF-α, aberrant mechanical loading, and metabolic stress in OA cartilage.

The three-gene panel and the nomogram showed exploratory discriminative performance across multiple independent cohorts, supporting their potential utility as tissue-level transcriptomic classifiers (59). The predicted miRNA-TF-mRNA regulatory network further provides a hypothesis-generating resource for putative upstream regulation; however, these regulatory relationships are computationally inferred and should be interpreted cautiously until validated experimentally.

Several analytic choices were made to balance coverage, robustness, and interpretability. HRGs were intentionally curated using an inclusive GeneCards keyword-based strategy to capture a broad Hb-associated annotation space at the systems level; consequently, the resulting signatures should be interpreted as Hb-associated rather than restricted to canonical globin genes. Outlier removal was restricted to WGCNA to improve network stability, whereas immune analyses were performed on the full merged dataset to preserve statistical power. In addition, quantile normalization was disabled in CIBERSORT to maintain compatibility with the merged, batch-corrected expression matrix across multiple microarray platforms.

This study also has limitations. First, it is retrospective and based on heterogeneous public datasets; although batch correction and external validation were performed, residual technical and clinical confounding cannot be excluded, and key covariates (e.g., age, sex, comorbidities, medication) were not consistently available. Second, the broad HRG definition may introduce annotation noise; future work could evaluate sensitivity using alternative curated heme/oxygen-related gene sets or by applying relevance-score thresholds. Third, the analyzed samples predominantly represent late-stage knee OA, and experimental validation was limited to mRNA-level changes in an IL-1β-stimulated immortalized C28/I2 chondrocyte line. C28/I2 cells do not fully reproduce the phenotype of primary human OA chondrocytes, and IL-1β stimulation captures only one inflammatory component of the OA microenvironment. Mechanical loading, cellular senescence, metabolic stress, matrix-remodeling conditions, and macrophage–chondrocyte interactions were not experimentally modeled. Further validation in primary OA and non-OA chondrocytes, cartilage explants, spatial transcriptomic datasets, and macrophage–chondrocyte co-culture systems will be important to determine whether LOXL1, THY1, and TYMS are causes, consequences, or markers of immune-associated remodeling.

## 5 Conclusion

In conclusion, this study identifies LOXL1, THY1, and TYMS as an annotation-derived Hb-associated immunotranscriptomic signature in OA cartilage. The three-gene panel was reproducibly upregulated across independent datasets and was associated with macrophage-/T-cell-related and antigen-presentation-related transcriptomic features. These findings support the potential tissue-level classification value of this signature and provide a basis for future validation using primary chondrocytes, single-cell/spatial approaches, and immune–chondrocyte interaction models.

## Data Availability

The original contributions presented in the study are included in the article/**Supplementary Material**. Further inquiries can be directed to the corresponding author.

## References

[1] Chen H Si L Hunter DJ Zhang L Chen ZS Wang X . Global and regional temporal changes in cross-country inequalities of site-specific osteoarthritis burden, 1990 to 2021. Arthritis Care Res (Hoboken). (2026) 78:215–26. doi: 10.1002/acr.25617 40755191 PMC12919705

[2] Korovina MO Valeeva AR Akhtyamov IF Brooks W Renaudineau Y Manukyan G . Joint tissues: Convergence and divergence of the pathogenetic mechanisms of rheumatoid arthritis and osteoarthritis. Int J Mol Sci. (2025) 26:8742. doi: 10.3390/ijms26178742 40943660 PMC12429403

[3] Thudium CS Hannani MT Collins JE Roemer FW Kraus VB Bihlet AR . Diagnosing, treating and monitoring the inflammatory endotype in osteoarthritis clinical trials. Expert Rev Mol Diagn. (2025) 25:709–22. doi: 10.1080/14737159.2025.2550639 40832883

[4] Mikulkova Z Gallo J Manukyan G Trajerova M Savara J Shrestha B . Complexity of synovial fluid-derived monocyte-macrophage-lineage cells in knee osteoarthritis. Cell Rep. (2024) 43:115011. doi: 10.1016/j.celrep.2024.115011 39661512

[5] Wu CJ Liu RX Huan SW Tang W Zeng YK Zhang JC . Senescent skeletal cells cross-talk with synovial cells plays a key role in the pathogenesis of osteoarthritis. Arthritis Res Ther. (2022) 24:59. doi: 10.1186/s13075-022-02747-4 35227288 PMC8883702

[6] Van Pevenage PM Birchmier JT June RK . Utilizing metabolomics to identify potential biomarkers and perturbed metabolic pathways in osteoarthritis: A systematic review. Semin Arthritis Rheum. (2023) 59:152163. doi: 10.1016/j.semarthrit.2023.152163 36736024 PMC9992342

[7] Feng P Lin Z-Y Liang M-J Zhang X-L Meng L-Q Duan T-T . Research progress on traditional Chinese medicine application in osteoarthritis. Tradit Med Res. (2025) 10:38–44. doi: 10.53388/tmr20240821001

[8] Zhang XA Kong H . Mechanism of HIFs in osteoarthritis. Front Immunol. (2023) 14:1168799. doi: 10.3389/fimmu.2023.1168799 37020556 PMC10067622

[9] Guo P Alhaskawi A Adel Abdo Moqbel S Pan Z . Recent development of mitochondrial metabolism and dysfunction in osteoarthritis. Front Pharmacol. (2025) 16:1538662. doi: 10.3389/fphar.2025.1538662 40017603 PMC11865096

[10] Zhu L Kamalathevan P Koneva LA Zarebska JM Chanalaris A Ismail H . Variants in ALDH1A2 reveal an anti-inflammatory role for retinoic acid and a new class of disease-modifying drugs in osteoarthritis. Sci Transl Med. (2022) 14:eabm4054. doi: 10.1126/scitranslmed.abm4054 36542696

[11] Zhang Z He T Gu H Zhao Y Tang S Han K . Single-cell RNA sequencing identifies the expression of hemoglobin in chondrocyte cell subpopulations in osteoarthritis. BMC Mol Cell Biol. (2024) 25:28. doi: 10.1186/s12860-024-00519-3 39736555 PMC11687149

[12] Kang X Zhang K Wang Y Zhao Y Lu Y . Single-cell RNA sequencing analysis of human chondrocytes reveals cell-cell communication alterations mediated by interactive signaling pathways in osteoarthritis. Front Cell Dev Biol. (2023) 11:1099287. doi: 10.3389/fcell.2023.1099287 37082621 PMC10112522

[13] Zhang F Zhang B Wang Y Jiang R Liu J Wei Y . An extra-erythrocyte role of haemoglobin body in chondrocyte hypoxia adaption. Nature. (2023) 622:834–41. doi: 10.1038/s41586-023-06611-6 37794190 PMC10600011

[14] Low JT Kim Y Matsushita M Ho PC . Role of oxygen sensing and hypoxia-inducible factors in orchestrating innate immune responses. Nat Immunol. (2025) 26:2138–47. doi: 10.1038/s41590-025-02317-1 41203908

[15] López M Gualillo O . Rheumatic diseases and metabolism: where centre and periphery meet. Nat Rev Rheumatol. (2024) 20:783–94. doi: 10.1038/s41584-024-01178-6 39478099

[16] Leach DT Stratton KG Irvahn J Richardson R Webb-Robertson BM Bramer LM . malbacR: A package for standardized implementation of batch correction methods for omics data. Anal Chem. (2023) 95:12195–9. doi: 10.1021/acs.analchem.3c01289 37551970

[17] Ritchie ME Phipson B Wu D Hu Y Law CW Shi W . limma powers differential expression analyses for RNA-sequencing and microarray studies. Nucleic Acids Res. (2015) 43:e47. doi: 10.1093/nar/gkv007 25605792 PMC4402510

[18] Safran M Rosen N Twik M BarShir R Stein TI Dahary D . The geneCards suite. In: Abugessaisa I Kasukawa T , editors. Practical Guide to Life Science Databases. Springer Nature Singapore, Singapore (2021). p. 27–56.

[19] Szklarczyk D Gable AL Lyon D Junge A Wyder S Huerta-Cepas J . STRING v11: protein-protein association networks with increased coverage, supporting functional discovery in genome-wide experimental datasets. Nucleic Acids Res. (2019) 47:D607–13. doi: 10.1093/nar/gky1131 30476243 PMC6323986

[20] Robin V Bodein A Scott-Boyer MP Leclercq M Périn O Droit A . Overview of methods for characterization and visualization of a protein-protein interaction network in a multi-omics integration context. Front Mol Biosci. (2022) 9:962799. doi: 10.3389/fmolb.2022.962799 36158572 PMC9494275

[21] Langfelder P Horvath S . WGCNA: an R package for weighted correlation network analysis. BMC Bioinf. (2008) 9:559. doi: 10.1186/1471-2105-9-559 19114008 PMC2631488

[22] Rezaie N Reese F Mortazavi A . PyWGCNA: a Python package for weighted gene co-expression network analysis. Bioinformatics. (2023) 39:btad415. doi: 10.1093/bioinformatics/btad415 37399090 PMC10359619

[23] Wei J Zhang J Wei J Hu M Chen X Qin X . Identification of AGXT2, SHMT1, and ACO2 as important biomarkers of acute kidney injury by WGCNA. PloS One. (2023) 18:e0281439. doi: 10.1371/journal.pone.0281439 36735737 PMC9897545

[24] Lin H Duan H Zheng J Jiang Z Xu Y Huang H . Clinical characteristics of thyroid eye disease and expression profile of peripheral blood immune cells. Sci Rep. (2025) 15:28666. doi: 10.1038/s41598-025-08904-4 40764715 PMC12325906

[25] Ashburner M Ball CA Blake JA Botstein D Butler H Cherry JM . Gene ontology: tool for the unification of biology. The Gene Ontology Consortium. Nat Genet. (2000) 25:25–9. doi: 10.1038/75556 10802651 PMC3037419

[26] Kanehisa M Goto S . KEGG: kyoto encyclopedia of genes and genomes. Nucleic Acids Res. (2000) 28:27–30. doi: 10.1093/nar/28.1.27 10592173 PMC102409

[27] Schriml LM Arze C Nadendla S Chang YW Mazaitis M Felix V . Disease Ontology: a backbone for disease semantic integration. Nucleic Acids Res. (2012) 40:D940–6. doi: 10.1093/nar/gkr972 22080554 PMC3245088

[28] Frost HR Amos CI . Gene set selection via LASSO penalized regression (SLPR). Nucleic Acids Res. (2017) 45:e114. doi: 10.1093/nar/gkx291 28472344 PMC5499546

[29] Yuan Y Fu M Li N Ye M . Identification of immune infiltration and cuproptosis-related subgroups in Crohn's disease. Front Immunol. (2022) 13:1074271. doi: 10.3389/fimmu.2022.1074271 36466876 PMC9713932

[30] Nedaie A Najafi AA . Support vector machine with Dirichlet feature mapping. Neural Netw. (2018) 98:87–101. doi: 10.1016/j.neunet.2017.11.006 29223012

[31] Claude E Leclercq M Thébault P Droit A Uricaru R . Optimizing hybrid ensemble feature selection strategies for transcriptomic biomarker discovery in complex diseases. NAR Genom Bioinform. (2024) 6:lqae079. doi: 10.1093/nargab/lqae079 38993634 PMC11237901

[32] Li J Zhou X Wen J Liu S Fan X . Establishment and validation of a nomogram clinical prediction model for osteoporosis in senile patients with type 2 diabetes mellitus. Sci Rep. (2024) 14:5343. doi: 10.1038/s41598-024-56127-w 38438532 PMC10912110

[33] Li T Lin C Zhao B Li Z Zhao Y Han X . Development and validation of a nomogram for predicting clinically relevant delayed gastric emptying in patients undergoing total pancreatectomy. BMC Surg. (2024) 24:283. doi: 10.1186/s12893-024-02575-0 39363181 PMC11448429

[34] Goldring MB Birkhead JR Suen LF Yamin R Mizuno S Glowacki J . Interleukin-1 beta-modulated gene expression in immortalized human chondrocytes. J Clin Invest. (1994) 94:2307–16. doi: 10.1172/jci117595 7989586 PMC330059

[35] Han J Zhan LN Huang Y Guo S Zhou X Kapilevich L . Moderate mechanical stress suppresses chondrocyte ferroptosis in osteoarthritis by regulating NF-κB p65/GPX4 signaling pathway. Sci Rep. (2024) 14:5078. doi: 10.1038/s41598-024-55629-x 38429394 PMC10907644

[36] Livak KJ Schmittgen TD . Analysis of relative gene expression data using real-time quantitative PCR and the 2-ΔΔCT method. Methods. (2001) 25:402–8. doi: 10.1006/meth.2001.1262 11846609

[37] Chou CH Shrestha S Yang CD Chang NW Lin YL Liao KW . miRTarBase update 2018: a resource for experimentally validated microRNA-target interactions. Nucleic Acids Res. (2018) 46:D296–302. doi: 10.1093/nar/gkx1067 29126174 PMC5753222

[38] Agarwal V Bell GW Nam JW Bartel DP . Predicting effective microRNA target sites in mammalian mRNAs. Elife. (2015) 4:e05005. doi: 10.7554/elife.05005 26267216 PMC4532895

[39] Hänzelmann S Castelo R Guinney J . GSVA: gene set variation analysis for microarray and RNA-seq data. BMC Bioinf. (2013) 14:7. doi: 10.1186/1471-2105-14-7 PMC3618321 23323831

[40] Zhanghuang C Yao Z Tang H Zhang K Wu C Li L . Identification of prognostic biomarkers in patients with Malignant rhabdoid tumor of the kidney based on mTORC1 signaling pathway-related genes. Front Mol Biosci. (2022) 9:843234. doi: 10.3389/fmolb.2022.843234 35558559 PMC9087638

[41] Newman AM Liu CL Green MR Gentles AJ Feng W Xu Y . Robust enumeration of cell subsets from tissue expression profiles. Nat Methods. (2015) 12:453–7. doi: 10.1038/nmeth.3337 25822800 PMC4739640

[42] Charoentong P Finotello F Angelova M Mayer C Efremova M Rieder D . Pan-cancer immunogenomic analyses reveal genotype-immunophenotype relationships and predictors of response to checkpoint blockade. Cell Rep. (2017) 18:248–62. doi: 10.1016/j.celrep.2016.12.019 28052254

[43] Robin X Turck N Hainard A Tiberti N Lisacek F Sanchez JC . pROC: an open-source package for R and S+ to analyze and compare ROC curves. BMC Bioinf. (2011) 12:77. doi: 10.1186/1471-2105-12-77 21414208 PMC3068975

[44] Jain L Bolam SM Monk AP Munro JT Chen E Tamatea J . Differential effects of hypoxia versus hyperoxia or physoxia on phenotype and energy metabolism in human chondrocytes from osteoarthritic compared to macroscopically normal cartilage. Int J Mol Sci. (2023) 24:7532. doi: 10.3390/ijms24087532 37108698 PMC10142591

[45] Arai Y Cha R Nakagawa S Inoue A Nakamura K Takahashi K . Cartilage homeostasis under physioxia. Int J Mol Sci. (2024) 25:9398. doi: 10.3390/ijms25179398 39273346 PMC11395513

[46] Sun Z Yan M Wang J Zhang H Ji X Xiao Y . Single-cell RNA sequencing reveals different chondrocyte states in femoral cartilage between osteoarthritis and healthy individuals. Front Immunol. (2024) 15:1407679. doi: 10.3389/fimmu.2024.1407679 38868774 PMC11167083

[47] García-Espinosa ME Limias-Quezada P Ortega-Meléndez AI Ballinas-Verdugo MA López-Gómez RE López-Espinosa E . Acupuncture-induced gene co-expression networks in postmenopausal women with osteoarthritis and osteoporosis: in-silico analysis. Acupuncture Herbal Med. (2024) 4:538–51. doi: 10.1097/hm9.0000000000000132 42348292

[48] Wang K Esbensen QY Karlsen TA Eftang CN Owesen C Aroen A . Low-input RNA-sequencing in patients with cartilage lesions, osteoarthritis, and healthy cartilage. Cartilage. (2021) 13:550s–62s. doi: 10.1177/19476035211057245 34775802 PMC8808811

[49] Newton MD Swahn H Orange DE Lesnak JB Price TJ Malfait AM . Cross-platform transcriptomic data integration identifies an overactive neuro-immune signature in human osteoarthritis synovium. Osteoarthritis Cartilage. (2025) 34:589–600. doi: 10.1016/j.joca.2025.08.013 40889615 PMC13112359

[50] Sunkara V Heinz GA Heinrich FF Durek P Mobasheri A Mashreghi MF . Combining segmental bulk- and single-cell RNA-sequencing to define the chondrocyte gene expression signature in the murine knee joint. Osteoarthritis Cartilage. (2021) 29:905–14. doi: 10.1016/j.joca.2021.03.007 33762205

[51] Kang H Strong AL Sun Y Guo L Juan C Bancroft AC . The HIF-1α/PLOD2 axis integrates extracellular matrix organization and cell metabolism leading to aberrant musculoskeletal repair. Bone Res. (2024) 12:17. doi: 10.1038/s41413-024-00320-0 38472175 PMC10933265

[52] Gordon JAR Evans MF Ghule PN Lee K Vacek P Sprague BL . Identification of molecularly unique tumor-associated mesenchymal stromal cells in breast cancer patients. PloS One. (2023) 18:e0282473. doi: 10.1371/journal.pone.0282473 36940196 PMC10027225

[53] Mimpen JY Hedley R Ridley A Baldwin MJ Windell D Bhalla A . Cellular characterisation of advanced osteoarthritis knee synovium. Arthritis Res Ther. (2023) 25:154. doi: 10.1186/s13075-023-03110-x 37612718 PMC10463598

[54] Pandey A Bhutani N . Profiling joint tissues at single-cell resolution: advances and insights. Nat Rev Rheumatol. (2024) 20:7–20. doi: 10.1038/s41584-023-01052-x 38057475 PMC11674069

[55] Sartori-Maldonado R Montaser H Soppa I Eurola S Juutila J Balaz M . Thymidylate synthase disruption to limit cell proliferation in cell therapies. Mol Ther. (2024) 32:2535–48. doi: 10.1016/j.ymthe.2024.06.014 38867450 PMC11405178

[56] White BS de Reyniès A Newman AM Waterfall JJ Lamb A Petitprez F . Community assessment of methods to deconvolve cellular composition from bulk gene expression. Nat Commun. (2024) 15:7362. doi: 10.1038/s41467-024-50618-0 39191725 PMC11350143

[57] Ferrao Blanco MN Bastiaansen-Jenniskens YM Chambers MG Pitsillides AA Narcisi R van Osch G . Effect of inflammatory signaling on human articular chondrocyte hypertrophy: potential involvement of tissue repair macrophages. Cartilage. (2021) 13:168s–74s. doi: 10.1177/19476035211021907 34165367 PMC8739598

[58] Henry ÓC O'Neill LAJ . Metabolic reprogramming in stromal and immune cells in rheumatoid arthritis and osteoarthritis: therapeutic possibilities. Eur J Immunol. (2025) 55:e202451381. doi: 10.1002/eji.202451381 40170391 PMC11962241

[59] Wu Y Huang HC Qin LX . Making external validation valid for molecular classifier development. JCO Precis Oncol. (2021) 5:1250–8. doi: 10.1200/po.21.00103 34377885 PMC8345919

