# Supplementary material for: LOXL1, THY1, and TYMS define an annotation-derived hemoglobin-associated immunotranscriptomic signature in osteoarthritis cartilage
Source: Front Immunol. 2026 Jul 9;17:1823558. doi: 10.3389/fimmu.2026.1823558 (PMC13391302; doi:10.3389/fimmu.2026.1823558)
Supplement: Supplementary file 2 [file SupplementaryFile2.docx]

Supplementary Material

## Supplementary Figure 1


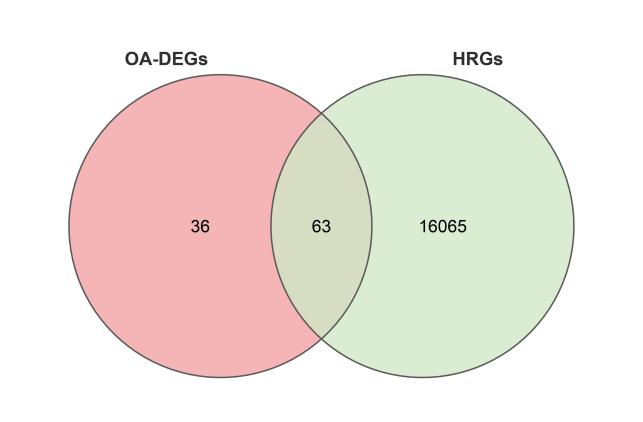
**Supplementary Figure 1.** Identification of HRDEGs. Venn diagram showing the overlap between OA-DEGs and HRGs.

## Supplementary Figure 2

**
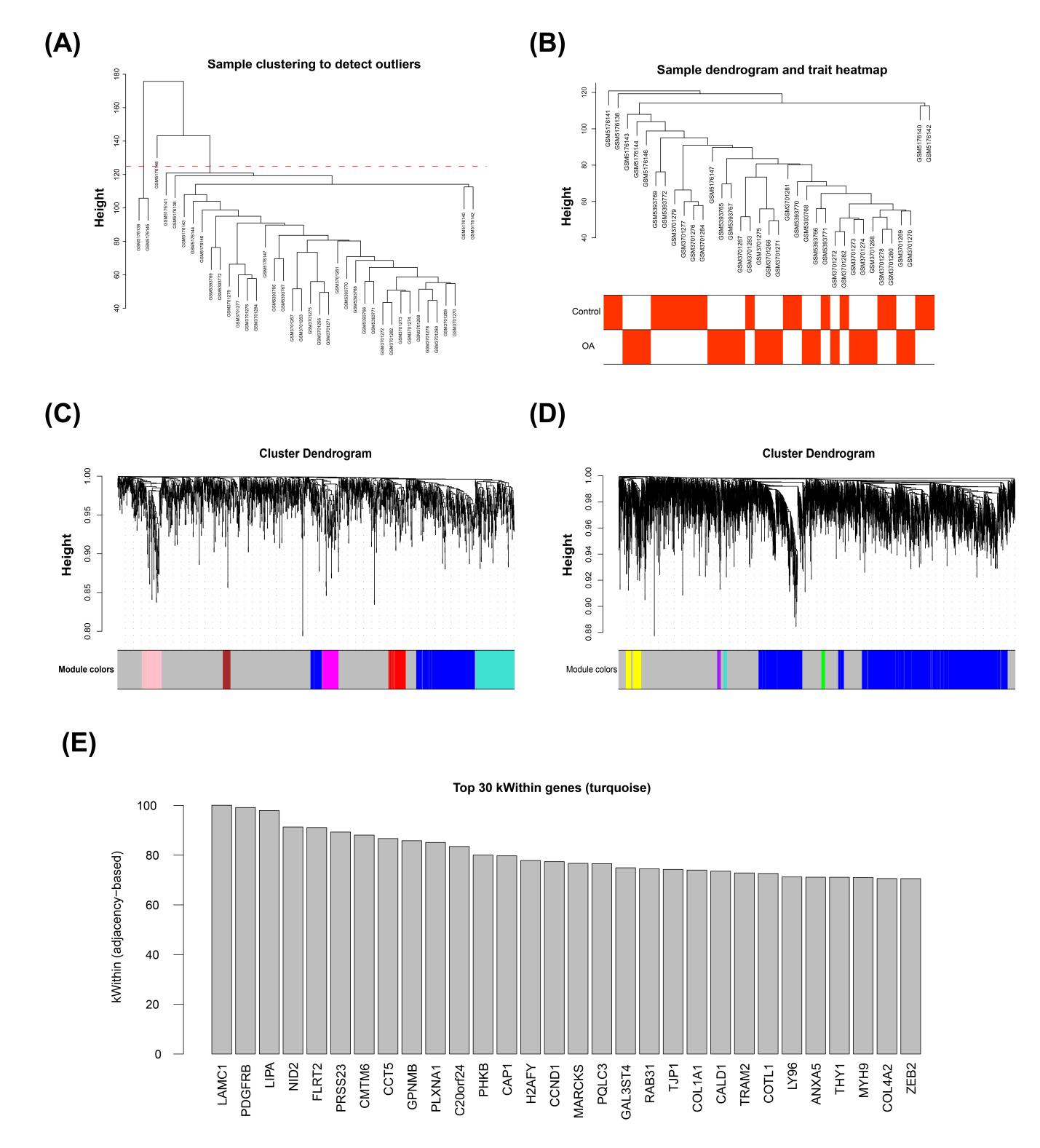
Supplementary Figure 2. Quality control and module detection in WGCNA.** (A) Sample clustering dendrogram used to detect outliers. (B) Sample dendrogram and trait heatmap showing OA and control status. (C, D) Additional gene dendrograms and module color assignments generated during WGCNA module detection. (E) Bar plot showing the top 30 genes ranked by intramodular connectivity (kWithin) in the turquoise module.

## Supplementary Figure 3


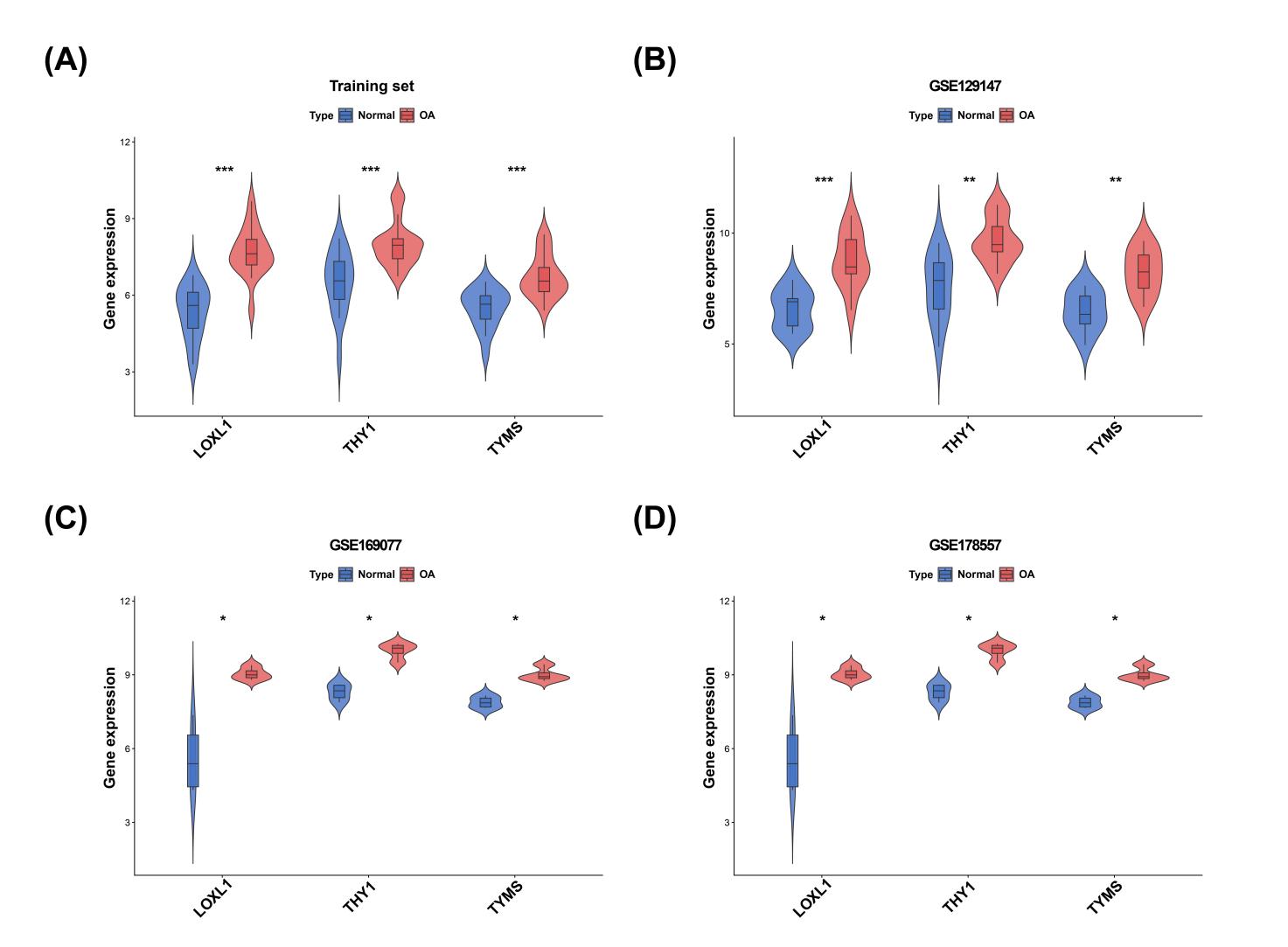
**Supplementary Figure 3. Expression of hub OA-HRDEGs in the training and validation datasets.** Violin plots showing expression levels of LOXL1, THY1, and TYMS in the training cohort (A), GSE129147 (B), GSE169077 (C), and GSE178557 (D).

- 1. **Supplementary Figure 4**


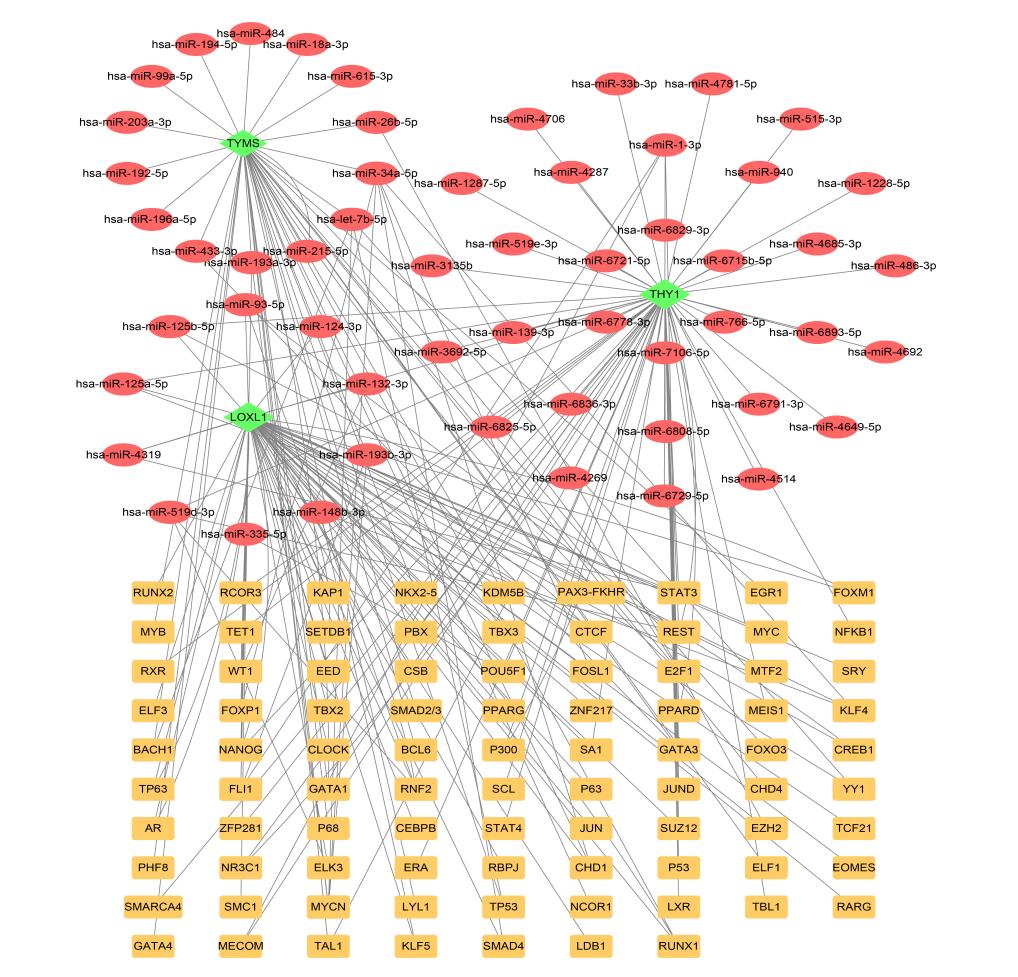


**Supplementary Figure 4. Complete predicted miRNA-TF-mRNA regulatory network of hub OA-HRDEGs.** The network includes 55 miRNAs, 88 transcription factors, three hub OA-HRDEGs, and 205 predicted regulatory interactions. Red nodes represent miRNAs, yellow nodes represent transcription factors, and green nodes represent hub OA-HRDEGs.
